# Cardiovascular outcomes between COVID-19 and non-COVID-19 pneumonia: a nationwide cohort study

**DOI:** 10.1186/s12916-023-03106-z

**Published:** 2023-10-20

**Authors:** Min-Taek Lee, Moon Seong Baek, Tae Wan Kim, Sun-Young Jung, Won-Young Kim

**Affiliations:** 1 Department of Global Innovative Drugs, The Graduate School of Chung-Ang University, Chung-Ang University, Seoul, Republic of Korea; 2 Division of Pulmonary and Critical Care Medicine, Department of Internal Medicine, Chung-Ang University Hospital, Chung-Ang University College of Medicine, Seoul, Republic of Korea; 3 College of Pharmacy, Chung-Ang University, Seoul, Republic of Korea

**Keywords:** Cardiovascular diseases, COVID-19, Hospitalization, Myocarditis, Pneumonia, Venous thrombosis

## Abstract

**Background:**

Previous studies that assessed the risk of cardiovascular outcomes in survivors of coronavirus disease 2019 (COVID-19) were likely limited by lack of generalizability and selection of controls nonrepresentative of a counterfactual situation regarding COVID-19-related hospitalization. This study determined whether COVID-19 hospitalization was associated with incident cardiovascular outcomes compared to non-COVID-19 pneumonia hospitalization.

**Methods:**

Nationwide population-based study conducted using the Korean National Health Insurance Service database. A cohort of 132,784 inpatients with COVID-19 (October 8, 2020–September 30, 2021) and a cohort of 31,173 inpatients with non-COVID-19 pneumonia (January 1–December 31, 2019) were included. The primary outcome was the major adverse cardiovascular event (MACE; a composite of myocardial infarction and stroke). Hazard ratios (HRs) with 95% confidence intervals (CIs) of all outcomes of interest were estimated between inverse probability of treatment-weighted patients with COVID-19 and non-COVID-19 pneumonia.

**Results:**

After weighting, the COVID-19 and non-COVID-19 pneumonia groups included 125,810 (mean [SD] age, 47.2 [17.6] years; men, 49.3%) and 28,492 patients (mean [SD] age, 48.6 [18.4] years; men, 47.2%), respectively. COVID-19 hospitalization was not associated with an increased risk of the MACE (HR, 0.84; 95% CI 0.69–1.03). However, the MACE (HR, 7.30; 95% CI 3.29–16.21), dysrhythmia (HR, 1.88; 95% CI 1.04–3.42), acute myocarditis (HR, 11.33; 95% CI 2.97–43.20), myocardial infarction (HR, 6.78; 95% CI 3.03–15.15), congestive heart failure (HR, 1.95; 95% CI 1.37–2.77), and thrombotic disease (HR, 8.26; 95% CI 4.06–16.83) risks were significantly higher in patients with COVID-19 aged 18–39 years. The findings were consistent after adjustment for preexisting cardiovascular disease. COVID-19 hospitalization conferred a higher risk of acute myocarditis (HR, 6.47; 95% CI 2.53–16.52) or deep vein thrombosis (HR, 1.97; 95% CI 1.38–2.80), regardless of vaccination status.

**Conclusions:**

Hospitalized patients with COVID-19 were not at an increased risk of cardiovascular outcomes compared to patients with non-COVID-19 pneumonia. Further studies are needed to evaluate whether the increased risk of cardiovascular outcomes is confined to younger patients.

**Supplementary Information:**

The online version contains supplementary material available at 10.1186/s12916-023-03106-z.

## Background

By September 6, 2023, the coronavirus disease 2019 (COVID-19) had affected more than 770 million people and caused over 6 million deaths worldwide [1]. Increasing levels of immunity and the development of novel therapies and vaccines have substantially reduced mortality rates. However, 6.2% of patients who recover from acute infection experience persistent symptoms, including fatigue, cognitive problems, or respiratory problems, known as post-COVID conditions [2].

The acute cardiovascular manifestations of COVID-19, which include thrombosis, dysrhythmia, heart failure, and shock, are associated with increased mortality [3]. Previous investigations of the risk of cardiovascular outcomes in the post-acute stage of COVID-19 in hospitalized patients were limited by inadequate risk adjustment for preexisting cardiovascular disease [4, 5]. In a cohort of 153,760 patients from the US Department of Veterans Affairs database, patients who survived acute COVID-19 were at increased risk of cardiovascular outcomes 1 year after infection, regardless of a history of cardiovascular disease or care setting (non-hospitalized, hospitalized, and intensive care unit admission), but the demographic composition (mostly White, older male) may limit the generalizability of the results and the COVID-19-specific sequelae were not assessed [6]. Hospitalization for pneumonia due to pathogens other than the severe acute respiratory syndrome coronavirus 2 (SARS-CoV-2) may confer a risk of long-term cardiovascular morbidity [7].

Using a Korean nationwide population-based cohort of patients without preexisting cardiovascular disease, we investigated the risk of cardiovascular outcomes among those who survived acute COVID-19 hospitalization. A cohort of hospitalized patients with non-COVID-19 pneumonia was used as a historical comparison group.

## Methods

### Data source

This retrospective cohort analysis collected data from the Korean National Health Insurance Service (NHIS) database, which includes claim codes from all citizens who reside in Korea, excluding medical aid beneficiaries and healthcare beneficiaries for veterans (~ 97% of the population in 2022) [8]. The database consists of detailed information regarding demographics; primary and secondary diagnoses; prescription and procedure charges; discharge status; and date of death. Diagnostic codes are based on the International Classification of Diseases, 10th Revision (ICD-10). The reimbursement scheme in Korea is based on fee-for-service and does not incentivize upcoding. All prescribed drugs were identified by Anatomical Therapeutic Chemical codes and the Korean Health Insurance Review and Assessment Service charge codes. The NHIS database was linked to the Korean nationwide COVID-19 registry constructed by the Korea Disease Control and Prevention Agency for confirming cases that involved COVID-19 diagnosis (positive real-time reverse transcription polymerase chain reaction from a nasopharyngeal swab) [9] and vaccination. All data were retrieved by an independent technician from the NHIS center.

The requirement for ethical approval was waived by the Institutional Review Board of Chung-Ang University due to analysis of de-identified patient records (1041078–202203-HR-086). The present study complied with the Strengthening the Reporting of Observational Studies in Epidemiology guidelines.

### Study population

The COVID-19 group comprised adults (age ≥ 18 years) with confirmed COVID-19 and who were hospitalized between October 8, 2020, and September 30, 2021. The index date was defined as the date of hospitalization. To ensure that the COVID-19 group had a similar follow-up duration as the control group, all patients were followed from the index date to the date of death or 3 months after the end of inclusion period, which was December 31, 2021.

The non-COVID-19 pneumonia group was constructed as a historical control group to (1) compare the risk of cardiovascular outcomes with those of COVID-19 hospitalization and (2) evaluate the sequelae specific to COVID-19. The group included adults (age ≥ 18 years) who were hospitalized for pneumonia caused by pathogens other than SARS-CoV-2 between January 1 and December 31, 2019. Additional file 1: Appendix 1 includes a detailed description of the types of pathogens and the ICD-10 codes. The last follow-up was on March 31, 2020.

For patients with multiple admissions, only the first admission was included. The exclusion criteria were (1) age < 18 years; (2) hospitalization prior to the index date; (3) preexisting cardiovascular disease identified using the ICD-10 codes within 1 year before the index date; and (4) death within the first 30 days following the index date. Preexisting cardiovascular diseases included stroke (ischemic or hemorrhagic); transient ischemic attack (TIA); atrial fibrillation; atrial flutter; ventricular arrhythmias; acute pericarditis; acute myocarditis; myocardial infarction; congestive heart failure; cardiac arrest; pulmonary embolism; and deep vein thrombosis (Additional file 2: Table S1).

### Data collection and definitions

Baseline covariates included age; sex; Charlson comorbidities [10] defined using claim codes within 1 year preceding the index date (Additional file 2: Table S2); income level; and hospital size. Body mass index (BMI) and smoking status were additionally retrieved in patients who had undergone a national health examination within 1 year before the index date. Cardiovascular drug therapies prescribed within 1 year before the index date were also extracted (Additional file 2: Table S3). Cardiovascular drug users were defined as patients who used any of the drugs above within 1–30 days before the index hospitalization, given that it was unlikely that these medications were discontinued within 1 month before the said hospitalization. The type of organ dysfunction was identified using the ICD-10 codes (Additional file 2: Table S4). Vasopressor use was defined as the infusion of norepinephrine, epinephrine, vasopressin, dopamine, or dobutamine during the index hospitalization. Procedure codes were retrieved to identify clinical care variables, including supplemental oxygen, high-flow nasal cannula, mechanical ventilation, renal replacement therapy, and extracorporeal membrane oxygenation (ECMO). Mild to moderate illness was defined as no oxygen therapy or receiving supplemental oxygen. Severe to critical illness was defined as receiving high-flow nasal cannula, mechanical ventilation, and/or ECMO.

### Outcomes

The primary outcome was the major adverse cardiovascular event (MACE; a composite of myocardial infarction and stroke) [11]. The secondary outcomes were all-cause mortality and each cardiovascular disease. For outcome variables, stroke, TIA, and myocardial infarction were identified using the combination of relevant ICD-10 codes and imaging [12] or procedure codes (Additional file 1: Appendix 2). The first incidence of any of the outcomes between 30 days after the index date and the end of follow-up was assessed. The first 30 days were excluded due to the complexity of differentiating outcomes as COVID-19 complications from the acute disease process.

### Statistical analysis

Data are reported as the mean (standard deviation, SD) or median (interquartile range, IQR) for continuous variables and as the frequency (percentage) for categorical variables. The occurrence of primary and secondary outcomes was presented as the cumulative incidence and incidence rates.

Propensity score (PS) analysis using inverse probability of treatment weighting (IPTW) was performed to adjust for potential confounding by baseline characteristics when comparing the COVID-19 and non-COVID-19 pneumonia groups. The PS for each patient was estimated using logistic regression model with COVID-19 as an independent variable and age, sex, comorbidities, income level, hospital size, cardiovascular drug therapies, organ dysfunction, vasopressor use, oxygen therapies, renal replacement therapy, and ECMO as covariates. Then, the PS was used to calculate the inverse probability weight as the probability of being in the COVID-19 group divided by 1 – the probability of being in the COVID-19 group. The IPTW creates a pseudo-population in which baseline covariates are balanced, meaning that both groups have similar proportions of covariates. The method has advantage of increasing power without changing the underlying estimates. To improve the precision of IPTW and reduce bias related to unmeasured confounders, 2.5% from each tail of the PS distribution was trimmed [13]. Covariate balance before and after weighting was assessed by standardized mean differences (SMDs). Hazard ratios (HRs) with 95% confidence intervals (CIs) of the incident primary and secondary outcomes between the groups were estimated from the Fine-Gray subdistribution hazard models where death was considered as a competing risk [14]. In addition, COVID-19 vaccination was employed as a time-varying covariate to eliminate any contribution of vaccine exposure to the potential risk of thrombosis, pericarditis, or myocarditis [15].

Subgroup analyses for the primary and secondary outcomes were conducted according to age (stratified by 18–39, 40–64, and ≥ 65 years); sex; diabetes; hypertension; dyslipidemia; Charlson Comorbidity Index; number of organ dysfunctions; and illness severity. Individual secondary outcomes were combined in a related category of composite outcome (e.g., stroke and TIA were combined to cerebrovascular disease). Multiple sensitivity analyses were performed to test the robustness of the main results. First, the analyses were re-run including patients with preexisting cardiovascular disease, who may be more vulnerable to COVID-19 complications. Second, the initial PS model did not include BMI and smoking status because these data were lacking in a considerable proportion of patients. Thus, a separate analysis investigated possible confounding by BMI and smoking status that included only patients who had these data. Third, some patients may have been diagnosed as stroke or TIA without brain imaging. Thus, the survival models with cerebrovascular outcomes identified using only the ICD-10 codes were utilized. Fourth, the associations between COVID-19 vaccination and MACE, myocardial infarction, stroke, and all-cause mortality were evaluated to assess if vaccines prevent cardiovascular outcomes.

No imputation strategy was conducted due to the small percentage of missing data (0.07%). All tests were two-tailed, and differences were considered statistically significant at a *P* value of < 0.05. All analyses were performed using SAS version 9.4 (SAS Institute, Cary, NC, USA).

## Results

### Study population

**Fig. 1:**
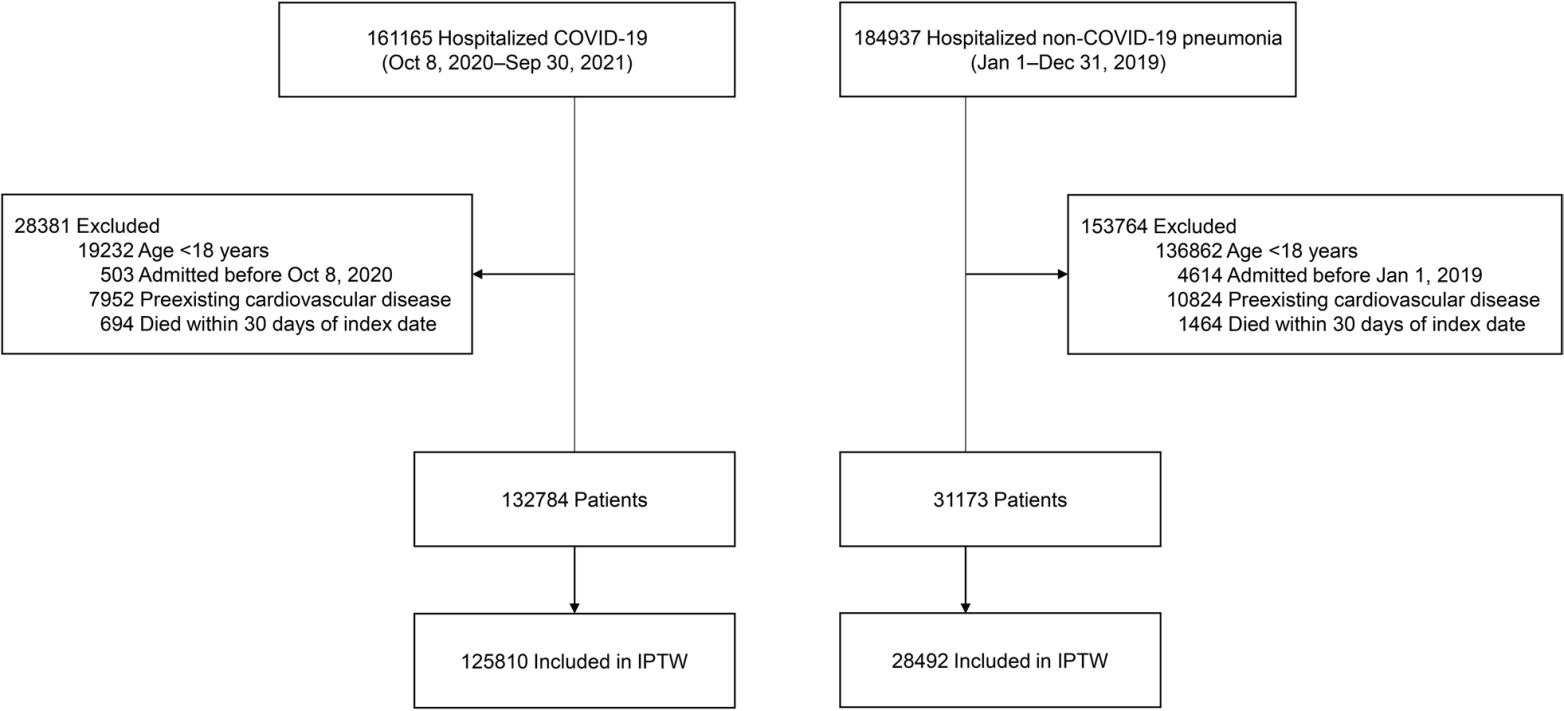
Cohort construction for the COVID-19 and non-COVID-19 pneumonia groups. COVID-19 coronavirus disease 2019, IPTW inverse probability of treatment weighting

During the study period, 161,165 individuals were hospitalized with COVID-19 (Fig. 1). After excluding those who met the exclusion criteria, 132,784 participants were included in the main analysis. In the control group, of the 184,937 patients with non-COVID-19 pneumonia who were hospitalized during the pre-COVID-19 period, 31,173 were included.

Patients hospitalized with COVID-19 were more likely to be younger, male, having less comorbidities, having higher income, and admitted in a higher volume hospital (Table 1). Patients with COVID-19 were less likely to have been prescribed with cardiovascular drug therapies. Moreover, the COVID-19 group less frequently developed organ dysfunction and received a lower intensity of organ support. The baseline characteristics and cardiovascular outcomes of study patients who died within the first 30 days following the index date are shown in Additional file 2: Tables S5 and S6. COVID-19 non-survivors were more likely to have cardiovascular risk factors, have been prescribed with cardiovascular drug therapies, develop organ dysfunction, and receive a higher intensity of organ support. However, the incidence of cardiovascular outcomes were generally comparable between the groups, except for the higher rates of atrial fibrillation (2.6% vs 1.4%) and deep vein thrombosis (3.5% vs 0.3%) in COVID-19 non-survivors.

**Table 1:** Baseline characteristics of participants in the COVID-19 and non-COVID-19 pneumonia groups

Characteristics	Before weighting			After weighting		
	COVID-19 (*n* = 132,784)	Non-COVID-19 pneumonia (*n* = 31,173)	SMD	COVID-19 (*n* = 125,810)	Non-COVID-19 pneumonia (*n* = 28,492)	SMD
Age, mean (SD), y	44.1 (15.7)	59.3 (20.0)	0.84	47.2 (17.6)	48.6 (18.4)	0.03
Sex, No. (%)			0.45			0.02
Male	68,553 (51.6)	13,622 (43.7)		61,964 (49.3)	13,436 (47.2)	
Female	64,231 (48.4)	17,551 (56.3)		63,845 (50.7)	15,056 (52.8)	
Comorbidities, No. (%)						
Diabetes	15,774 (11.9)	7777 (25.0)	–0.34	17,661 (14.0)	4361 (15.3)	–0.04
Hypertension	22,430 (16.9)	11,362 (36.5)	–0.45	24,868 (19.8)	5904 (20.7)	–0.02
Dyslipidemia	31,279 (23.6)	11,891 (38.2)	–0.32	32,811 (26.1)	8241 (28.9)	–0.06
Peripheral vascular disease	6584 (5.0)	4293 (13.8)	–0.31	7908 (6.3)	1901 (6.7)	–0.02
Chronic pulmonary disease	16,694 (12.6)	16,431 (52.7)	–0.95	23,791 (18.9)	5717 (20.1)	–0.03
Chronic liver disease	18,244 (13.7)	7342 (23.6)	–0.25	19,545 (15.5)	4943 (17.4)	–0.05
Chronic kidney disease	687 (0.5)	509 (1.6)	–0.11	796 (0.6)	215 (0.8)	–0.01
Malignancy	4688 (3.5)	3310 (10.6)	–0.28	5625 (4.5)	1439 (5.1)	–0.03
Charlson Comorbidity Index, mean (SD)	0.8 (1.2)	2.2 (1.7)	0.84	1.1 (1.5)	1.2 (1.4)	0.05
Income level, No. (%)			0.17			0.03
Q1 (lowest)	35,243 (26.5)	8032 (25.8)		33,568 (26.7)	7634 (26.8)	
Q2	31,560 (23.8)	8554 (27.4)		31,040 (24.7)	6942 (24.4)	
Q3	34,134 (25.7)	8118 (26.0)		32,909 (26.2)	7544 (26.5)	
Q4 (highest)	31,189 (23.5)	6265 (20.1)		27,644 (22.0)	6229 (21.9)	
Missing	658 (0.5)	204 (0.7)		648 (0.5)	143 (0.5)	
Hospital size, No. (%)			0.35			0.09
< 500 beds	97,822 (73.7)	22,369 (71.8)		94,921 (75.5)	21,727 (76.3)	
≥ 500 beds	33,917 (25.5)	6359 (20.4)		29,381 (23.4)	6333 (22.2)	
Missing	1045 (0.8)	2445 (7.8)		1508 (1.2)	432 (1.5)	
Cardiovascular drug therapy, No. (%)						
ACE inhibitor	17 (0.01)	20 (0.1)	–0.03	25 (0.02)	9 (0.03)	–0.01
ARB	409 (0.3)	320 (1.0)	–0.09	532 (0.4)	159 (0.6)	–0.02
Beta-blocker	703 (0.5)	336 (1.1)	–0.06	772 (0.6)	197 (0.7)	–0.01
Calcium-channel blocker	761 (0.6)	683 (2.2)	–0.14	1014 (0.8)	287 (1.0)	–0.02
Diuretic	543 (0.4)	721 (2.3)	–0.16	755 (0.6)	256 (0.9)	–0.03
Statin	378 (0.3)	368 (1.2)	–0.11	508 (0.4)	170 (0.6)	–0.03
Insulin	482 (0.4)	406 (1.3)	–0.10	623 (0.5)	204 (0.7)	–0.03
Other hypoglycemic agents	570 (0.4)	508 (1.6)	–0.12	790 (0.6)	256 (0.9)	–0.03
Antiplatelet agents	411 (0.3)	390 (1.3)	–0.11	556 (0.4)	175 (0.6)	–0.02
Organ dysfunction, No. (%)						
Cardiovascular	1412 (1.1)	1806 (5.8)	–0.26	1856 (1.5)	584 (2.1)	–0.04
Respiratory	15,931 (12.0)	8377 (26.9)	–0.38	16,588 (13.2)	4129 (14.5)	–0.04
Neurologic	407 (0.3)	543 (1.7)	–0.14	546 (0.4)	173 (0.6)	–0.02
Hematologic	1747 (1.3)	202 (0.6)	0.07	801 (0.6)	219 (0.8)	–0.02
Hepatic	34 (0.03)	54 (0.2)	–0.05	44 (0.3)	11 (0.04)	–0.002
Renal	328 (0.2)	812 (2.6)	–0.20	484 (0.4)	161 (0.6)	–0.03
Metabolic	52 (0.04)	58 (0.2)	–0.04	72 (0.1)	22 (0.1)	–0.01
Vasopressor use, No. (%)	1302 (1.0)	1731 (5.6)	–0.26	1741 (1.4)	547 (1.9)	–0.04
Oxygen therapy, No. (%)						
No oxygen	116,873 (88.0)	22,893 (73.4)	0.38	109,258 (86.8)	24,400 (85.6)	0.03
Supplemental oxygen	15,784 (11.9)	8245 (26.5)	–0.38	16,395 (13.0)	4075 (14.3)	–0.04
High-flow nasal cannula	3051 (2.3)	841 (2.7)	–0.03	2217 (1.8)	669 (2.4)	–0.04
Mechanical ventilation	859 (0.6)	744 (2.4)	–0.14	1020 (0.8)	327 (1.1)	–0.03
Renal replacement therapy, No. (%)	167 (0.1)	401 (1.3)	–0.14	239 (0.2)	88 (0.3)	–0.02
ECMO, No. (%)	62 (0.1)	15 (0.1)	–0.001	42 (0.03)	14 (0.1)	–0.01

*ACE* Angiotensin-converting enzyme, *ARB* Angiotensin II receptor blocker, *COVID-19* coronavirus disease 2019, *ECMO* Extracorporeal membrane oxygenation, *SMD* Standardized mean difference

### Incident primary and secondary outcomes in COVID-19 vs non-COVID-19 pneumonia

After weighting, the COVID-19 and non-COVID-19 pneumonia groups included 125,810 (mean [SD] age, 47.2 [17.6] years; men, 49.3%) and 28,492 patients (mean [SD] age, 48.6 [18.4] years; men, 47.2%), respectively (Table 1). SMDs in baseline characteristics between the groups were all less than 0.10, indicating that covariates were well-balanced. The median (IQR) follow-up in the COVID-19 and non-COVID-19 pneumonia groups was 169 (128–287) and 277 (142–376) days, respectively. The COVID-19 and non-COVID-19 pneumonia groups had 26,106,978 and 7,589,952 person-days of follow-up, respectively.

**Fig. 2:**
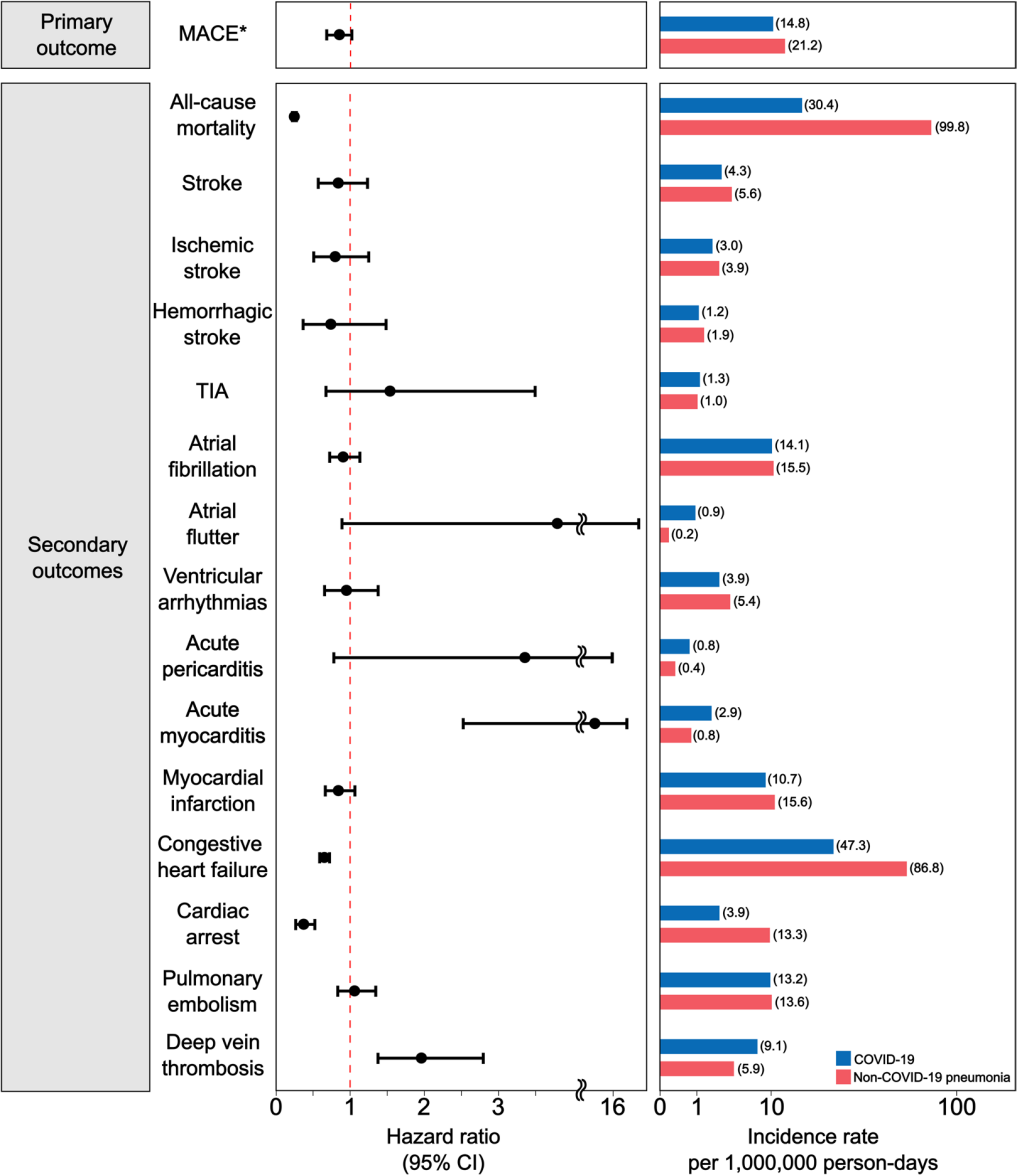
Risk of cardiovascular outcomes in participants hospitalized for COVID-19 or non-COVID-19 pneumonia. The hazard ratios and 95% CIs have been estimated in the inverse probability of treatment-weighted COVID-19 (*n* = 125,810) and non-COVID-19 pneumonia (*n* = 28,492) groups. The length of the bar represents the incidence rates per 1,000,000 person-days. CI confidence interval, COVID-19 coronavirus disease 2019, MACE major adverse cardiovascular event, TIA transient ischemic attack. * A composite of myocardial infarction and stroke

**Fig. 3:**
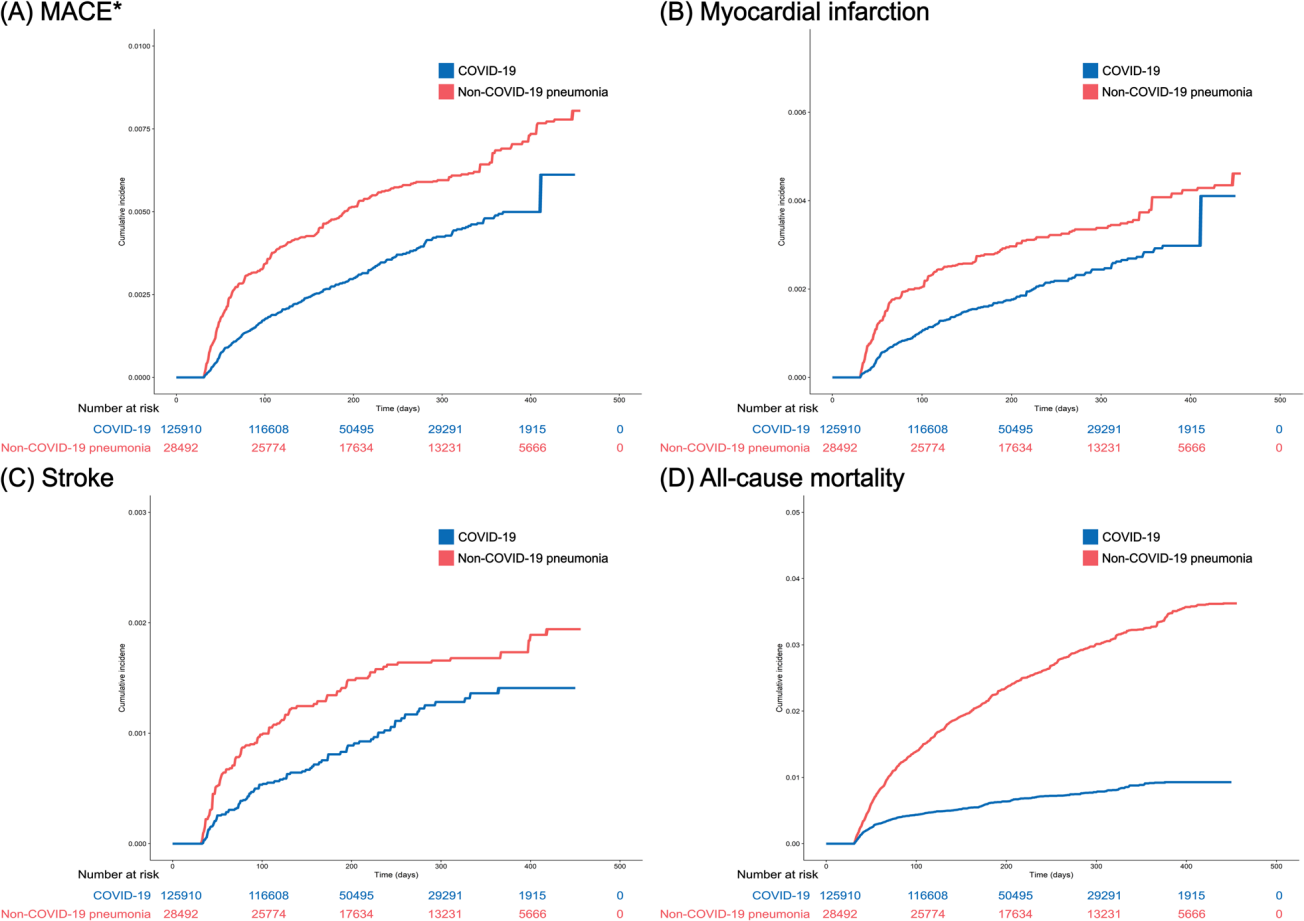
Cumulative incidence of **A** MACE, **B** myocardial infarction, **C** stroke, and **D** all-cause mortality in the COVID-19 and non-COVID-19 pneumonia groups. The median (interquartile range) time to MACE was 91 (53–156) and 76 (46–162) days in the COVID-19 and non-COVID-19 pneumonia groups, respectively. COVID-19 coronavirus disease 2019, MACE major adverse cardiovascular event. * A composite of myocardial infarction and stroke

Overall, the incidence rate of MACE (per 1,000,000 person-days) was 14.8 and 21.2 among patients who survived the first 30 days of COVID-19 and non-COVID-19 pneumonia hospitalization, respectively (Fig. 2 and Additional file 2: Table S7). The risk was not significantly higher in the COVID-19 group (HR, 0.84; 95% CI 0.69–1.03). The cumulative incidence of MACE, myocardial infarction, stroke, and all-cause mortality in both groups are shown in Fig. 3. The all-cause mortality, congestive heart failure, and cardiac arrest risks were significantly lower in the COVID-19 group (Fig. 2 and Additional file 2: Table S7). Conversely, patients who survived acute COVID-19 hospitalization presented with a significantly higher risk of acute myocarditis (HR, 6.47; 95% CI 2.53–16.52) or deep vein thrombosis (HR, 1.97; 95% CI 1.38–2.80).

### Subgroup analyses

**Fig. 4:**
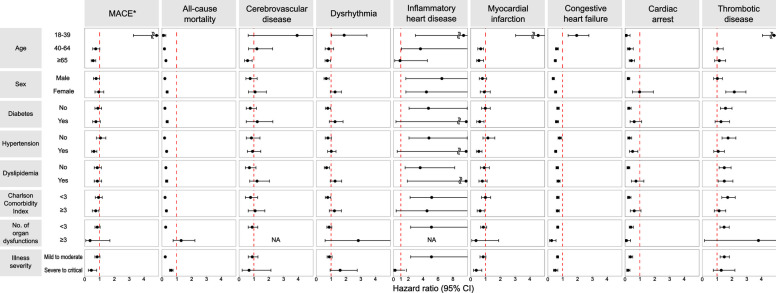
Subgroup analyses comparing the risk of cardiovascular outcomes in participants hospitalized for COVID-19 or non-COVID-19 pneumonia. The hazard ratios and 95% CIs have been estimated in the inverse probability of treatment-weighted COVID-19 (*n* = 125,810) and non-COVID-19 pneumonia (*n* = 28,492) groups. *CI* confidence interval, *COVID-19* coronavirus disease 2019, *MACE* major adverse cardiovascular event. ^*^ A composite of myocardial infarction and stroke

The results of subgroup analyses were generally consistent with those in the primary analysis, although some did not reach statistical significance (Fig. 4 and Additional file 2: Table S8). Notably, the MACE (HR, 7.30; 95% CI 3.29–16.21), dysrhythmia (HR, 1.88; 95% CI 1.04–3.42), acute myocarditis (HR, 11.33; 95% CI 2.97–43.20), myocardial infarction (HR, 6.78; 95% CI 3.03–15.15), congestive heart failure (HR, 1.95; 95% CI 1.37–2.77), and thrombotic disease (HR, 8.26; 95% CI 4.06–16.83) risks were significantly higher in patients with COVID-19 aged 18–39 years. The risk of acute myocarditis was also significantly higher in COVID-19 patients who had no cardiovascular risk factors, less comorbidities, less organ dysfunction, and mild to moderate illness. Higher HR of acute myocarditis was observed in male compared to female patients. For thrombotic disease, the risk was significantly higher in female patients with COVID-19 who had no cardiovascular risk factors, less comorbidities, less organ dysfunction, and mild to moderate illness.

### Sensitivity analyses

First, for the analyses including patients with preexisting cardiovascular disease, baseline comorbidities, cardiovascular drug therapies, organ dysfunction, and organ support were more prevalent than those in the primary cohort (Additional file 2: Table S9). The risks of the most cardiovascular outcomes were significantly lower in the COVID-19 group (Additional file 2: Table S10 and Additional file 3: Fig. S1), although the risk of acute myocarditis (HR, 1.99; 95% CI 1.09–3.64) and deep vein thrombosis (HR, 0.61; 95% CI 0.51–0.73) in the COVID-19 group were attenuated after adjustment for preexisting cardiovascular disease. Similar to the primary cohort, the risks of MACE, acute myocarditis, myocardial infarction, and thrombotic disease were significantly higher in patients with COVID-19 aged 18–39 years, although HRs decreased compared to those in the primary cohort (Additional file 2: Table S11 and Additional file 3: Fig. S2). Second, the PS model with available BMI and smoking status data constructed a cohort with similar baseline characteristics compared to the primary cohort (Additional file 2: Table S12). Subgroup analyses with stratification according to BMI and smoking status showed no significant interaction with any variable (Additional file 2: Table S13 and Additional file 3: Fig. S3). Third, the results of the analyses were consistent when only the ICD-10 code-based cerebrovascular outcomes were included (Additional file 2: Table S14). Fourth, the risks of MACE, myocardial infarction, stroke, and all-cause mortality increased in COVID-19 patients who were not vaccinated (Additional file 3: Fig. S4).

## Discussion

This large-scale, population-based study of 154,302 Korean patients did not reveal a higher incidence of post-acute cardiovascular outcomes in patients with COVID-19 hospitalization compared to those hospitalized with non-COVID-19 pneumonia. The findings were consistent after adjustment for preexisting cardiovascular disease. However, the risks were higher in patients with COVID-19 aged 18–39 years. COVID-19 patients had a higher risk of acute myocarditis or deep vein thrombosis, regardless of vaccination status. The risks were more pronounced among younger patients with less comorbidities and illness severity. Finally, the current results suggest that COVID-19 vaccination may prevent cardiovascular outcomes.

Several studies from the large US, UK, or global database have provided substantial evidence that the risk of cardiovascular outcomes was significantly higher in COVID-19 survivors compared to non-COVID-19 controls [6, 16, 17]. COVID-19 is associated with endothelial dysfunction and a prothrombic state [18], which can result in micro- and macrovascular thrombosis with subsequent injury to various organs [19]. Furthermore, putative mechanisms include fibrosis and scarring of cardiac tissue due to increased proinflammatory cytokine secretion and activation of transforming growth factor-β signaling and metabolic changes that favor viral survival, resulting in persistent inflammation and tissue injury [20, 21].

Despite these mechanisms, COVID-19 hospitalization was not associated with the increased risk of cardiovascular outcomes in the present study. There are several explanations for these observations. First, a weighted control group was selected from hospitalized patients with non-COVID-19 pneumonia. This study design was based on the mechanisms that the risk of cardiovascular disease may be increased by proinflammatory changes of the atherosclerotic lesions [22], persistent inflammation [23], and persistent procoagulant state [24], which can be observed in non-COVID-19 pneumonia. Comparable rates of organ dysfunction and intensity of organ support were found between the COVID-19 and non-COVID-19 pneumonia groups. Alternatively, previous studies compared outcomes after COVID-19 and general hospital admission [6, 16, 17], which lacks adequate consideration of differential risk by pneumonia severity, and thereby does not necessarily represent an appropriate counterfactual situation to COVID-19 hospitalization. Second, there may be geographic variations in the prevalence of COVID-19 and cardiovascular disease; most previous studies evaluated cardiovascular outcomes in the Western countries. The current study in an Asian population may be clinically relevant, given that angiotensin-converting enzyme 2 expression in tissues differs in the East Asian populations compared to other non-Asian populations under similar conditions [25].

The present study excluded patients who died or had any cardiovascular outcomes within the first 30 days of hospital admission, because cardiac injury is often associated with multiorgan dysfunction during critical illness rather than the disease itself [26]. This method may lead to an underestimation of the true risk of cardiovascular outcomes among patients with COVID-19. For instance, recent studies have shown that cardiovascular complications often preceded death in non-survivors, compared to patients who survived, and most events occurred within 30 days of infection [16, 27]. However, the incidence of outcomes were generally comparable between non-survivors with COVID-19 and non-COVID-19 pneumonia, although the COVID-19 group were more likely to have cardiovascular risk factors and greater illness severity. Moreover, the median time to MACE was longer than 30 days in both groups.

The finding that the increased risk of cardiovascular outcomes is confined to younger patients (aged 18–39 years), who are less likely to have comorbidities and classic cardiovascular risk factors, implies that COVID-19 may be an independent risk factor in this subgroup. Furthermore, the risks were attenuated after adjustment for preexisting cardiovascular disease. However, these should be interpreted with caution due to relatively small number of events producing wide CIs. Acute myocarditis associated with COVID-19 can present as a direct cardiac injury mediated by SARS-CoV-2, which could trigger a nonspecific inflammation using molecular mimicry between viral proteins and cardiomyocytes [28, 29]. The current finding that the risk of acute myocarditis was significantly higher in the COVID-19 group suggest that myocarditis may be triggered by SARS-CoV-2 independently of the occurrence of pneumonia. Furthermore, the increased risk of acute myocarditis, regardless of vaccination status, among younger COVID-19 patients with no cardiovascular comorbidities and less illness severity supports the notion that SARS-CoV-2 may act as an independent risk factor. Notably, the risk of acute myocarditis was more pronounced among COVID-19 patients without preexisting cardiovascular disease. A higher risk of acute myocarditis was observed in male COVID-19 patients, which contrasts with the Western studies showing that female COVID-19 survivors had a higher risk of myocarditis [17]. The risk of deep vein thrombosis was significantly higher in the COVID-19 group without preexisting cardiovascular disease, and this observation is consistent with the results of previous studies [5, 6, 16, 30]. Finally, the finding that vaccination against COVID-19 was associated with a decreased risk of cardiovascular outcomes is consistent with previous studies supporting vaccination, especially for patients with preexisting cardiovascular disease [12].

To the best of our knowledge, this is the first study to assess whether COVID-19 hospitalization was associated with cardiovascular outcomes compared to hospitalization for pneumonia due to other respiratory pathogens in a nationwide cohort of Asian patients. The study derived its cohorts from an entire population in Korea to increase generalizability and had sufficient power to quantify multiple and rare outcomes overall and across various subgroups. In addition, all available baseline covariates were included in the PS used for weighting, and the primary results were generally consistent with multiple sensitivity analyses. Previous studies may have misclassified individuals due to the inherent limitations of using claims data to define variables. For instance, some COVID-19 patients might have been enrolled in the control group if they did not have enough symptoms to be tested. To overcome this limitation, controls were recruited from patients hospitalized before the pandemic (January 1–December 31, 2019) in the present study. Cerebrovascular outcomes and myocardial infarction were rigorously defined using the ICD-10 codes and related imaging or procedures rather than just the codes to obtain more reliable results.

The current study has several limitations. First, the retrospective observational design precludes any causal inference regarding the associations between COVID-19, non-COVID-19 pneumonia, and cardiovascular outcomes. Moreover, there were differences in baseline characteristics between the groups. A robust model was used to reduce the effect of such differences, but the possibility of unmeasured confounders exists. Second, the accuracy of the ICD-10 codes may be limited. It is possible that all ICD-10 codes for each outcome might not have been captured, although the most common ones were included. In addition, patients who had asymptomatic infections or resided in nursing homes for palliative care who were not hospitalized may also develop post-acute complications but were not included. Meanwhile, this approach might have described a more specific population of severe pneumonia. Third, the database did not include information regarding vital signs and laboratory data, but diagnoses, prescriptions, and procedures were used as surrogates of pneumonia severity. Fourth, the diagnosis of acute myocarditis was not supported by histological evidence or cardiac magnetic resonance imaging [31, 32]. Furthermore, a very small number of events (< 100) may be a limiting factor in analysis. Fifth, the increased risk of acute myocarditis in COVID-19 patients might be a result of increased medical care after SARS-CoV-2 infection. However, this ascertainment bias is unlikely to fully explain the present results because the risk of other cardiovascular outcomes was not higher in the COVID-19 group. Sixth, the cardiovascular manifestations of COVID-19 may change over time with the emergence of new variants that induce varying degrees of severity [33]. However, a subgroup analysis assessing the risk of cardiovascular outcomes according to different SARS-CoV-2 variants was not feasible due to lack of data. Seventh, unequal follow-up in the study groups, probably due to differing distribution of the index dates, may bias the analysis. In addition, the follow-up time was relatively short, although the median time to MACE was less than 100 days for both the COVID-19 and non-COVID-19 pneumonia groups.

## Conclusions

Korean patients who survived acute COVID-19 hospitalization were not at increased risk of cardiovascular outcomes than those who were hospitalized with non-COVID-19 pneumonia. The present results indicate that the size and direction of the estimates may depend on the choice of control admissions. The findings that the risk of acute myocarditis or deep vein thrombosis was higher in the COVID-19 group are consistent with previous studies suggesting clinically at-risk population. The increased risk of cardiovascular outcomes in younger patients with COVID-19 warrants further study.

### Supplementary Information

**Additional file 1:** **Appendix 1.** International Classification of Diseases, 10th Revision codes used for the identification of pneumonia due to pathogens other than the severe acute respiratory syndrome coronavirus 2. **Appendix 2.** Outcome definitions.

**Additional file 2:** **Table S1.** Coding for cardiovascular diseases. **Table S2.** Comorbidities based on the Charlson Comorbidity Index. **Table S3.** Types and codes for cardiovascular drug therapies.**Table S4.** ICD-10-based classification of organ dysfunction. **Table S5.** Baseline characteristics of participants in the COVID-19 and non-COVID-19 pneumonia groups who died within the first 30 days following the index date. **Table S6.** Cardiovascular outcomes of participants in the COVID-19 and non-COVID-19 pneumonia groups who died within the first 30 days following the index date. **Table S7.** Comparison of the risk of cardiovascular outcomes in participants hospitalized for COVID-19 or non-COVID-19 pneumonia. **Table S8.** Subgroup analyses of the risk of cardiovascular outcomes in participants hospitalized for COVID-19 or non-COVID-19 pneumonia. **Table S9.** Baseline characteristics of participants in the COVID-19 and non-COVID-19 pneumonia groups, including patients with preexisting cardiovascular disease. **Table S10.** Comparison of the risk of cardiovascular outcomes in participants hospitalized for COVID-19 or non-COVID-19 pneumonia, in cohorts including patients with preexisting cardiovascular disease. **Table S11.** Subgroup analyses of the risk of cardiovascular outcomes in participants hospitalized for COVID-19 or non-COVID-19 pneumonia, in cohorts including patients with preexisting cardiovascular disease. **Table S12.** Baseline characteristics of participants in the COVID-19 and non-COVID-19 pneumonia groups, including only patients with body mass index and smoking status data. **Table S13.** Subgroup analyses of the risk of cardiovascular outcomes in participants hospitalized for COVID-19 or non-COVID-19 pneumonia, in cohorts that included only patients with body mass index and smoking status data. **Table S14.** Comparison of the risk of cerebrovascular outcomes, identified using only the International Classification of Diseases, 10th Revision codes, in participants hospitalized for COVID-19 or non-COVID-19 pneumonia. 

**Additional file 3:** **Fig. S1.** Risk of cardiovascular outcomes in participants hospitalized for COVID-19 or non-COVID-19 pneumonia, in cohorts that included patients with preexisting cardiovascular disease. **Fig. S2.** Subgroup analyses comparing the risk of cardiovascular outcomes in participants hospitalized for COVID-19 or non-COVID-19 pneumonia, in cohorts that included patients with preexisting cardiovascular disease. **Fig. S3.** Subgroup analyses comparing the risk of cardiovascular outcomes in participants hospitalized for COVID-19 or non-COVID-19 pneumonia, in cohorts that included only patients with body mass index and smoking status data. **Fig. S4.** Cumulative incidence of (A) MACE, (B) myocardial infarction, (C) stroke, and (D) all-cause mortality in the COVID-19 group stratified according to vaccination status.

## Data Availability

All data generated or analyzed during this study are included in this published article and its supplementary information files.

## Abbreviations

BMI: Body mass index
CI: Confidence interval
COVID-19: Coronavirus disease 2019
ECMO: Extracorporeal membrane oxygenation
HR: Hazard ratio
ICD: International Classification of Diseases
IPTW: Inverse probability of treatment weighting
IQR: Interquartile range
MACE: Major adverse cardiovascular event
NHIS: National Health Insurance Service
PS: Propensity score
SARS-CoV-2: Severe acute respiratory syndrome coronavirus 2
SD: Standard deviation
SMD: Standardized mean difference
TIA: Transient ischemic attack
