# Supplementary material for: Cardiovascular outcomes between COVID-19 and non-COVID-19 pneumonia: a nationwide cohort study
Source: BMC Med. 2023 Oct 20;21:394. doi: 10.1186/s12916-023-03106-z (PMC10588072; doi:10.1186/s12916-023-03106-z)
Supplement: Supplementary file 1 — Additional file 1: Appendix 1. International Classification of Diseases, 10th Revision codes used for the identification of pneumonia due to pathogens other than the severe acute respiratory syndrome coronavirus 2. Appendix 2. Outcome definitions. [file 12916_2023_3106_MOESM1_ESM.docx]

**Appendix 1** International Classification of Diseases, 10th Revision codes used for the identification of pneumonia due to pathogens other than the severe acute respiratory syndrome coronavirus 2

J09, Influenza due to identified zoonotic or pandemic influenza virus; J10, Influenza due to identified seasonal influenza virus; J11, Influenza, virus not identified; J12, Viral pneumonia, not elsewhere classified (NEC); J13, Pneumonia due to *Streptococcus pneumoniae*; J14, Pneumonia due to *Haemophilus influenzae*; J15, Bacterial pneumonia, NEC; J16, Pneumonia due to other infectious organisms, NEC; J17, Pneumonia in diseases classified elsewhere; J18, Pneumonia, organism unspecified; A202, Pneumonic plague; B012, Varicella pneumonia; B485, Pneumocystosis; B250, Cytomegaloviral pneumonitis; B052, Measles complicated by pneumonia; J851, Abscess of lung with pneumonia; B206, Human immunodeficiency virus disease resulting in *Pneumocystis jirovecii* pneumonia

**Appendix 2** Outcome definitions

Stroke or transient ischemic attack was defined using the International Classification of Diseases, 10th Revision (ICD-10) codes and the related brain imaging (e.g., computed tomography or magnetic resonance imaging) findings.

| Variable | ICD-10 or EDI codes |
| --- | --- |
| Stroke |  |
| Ischemic | I63 |
| Hemorrhagic | I60, I61, I62 |
| Ischemic or hemorrhagic | I64 |
| TIA | G45 |
| Brain imaging | HA441, HA451, HA461, HA471, HA511, HA521, HA531, HA551, HA561, HA851, HI101, HI201, HI301, HJ401, HI501, HI535, HJ101, HJ201, HJ301, HJ401, HJ501, HJ535, HF101, HF102, HF201, HF202, HE101, HE201, HE301, HE401, HE501, HE135, HE235, HE535 |

*EDI* Electronic Data Interchange, *ICD* International Classification of Diseases, *TIA* transient ischemic attack

Myocardial infarction was defined based on claims that included ICD-10 codes, or if percutaneous intervention and/or coronary artery bypass graft were performed.

| Variable | ICD-10 or EDI codes |
| --- | --- |
| Myocardial infarction | I21 |
| Percutaneous intervention | M655x–M657x |
| Coronary artery bypass graft | OA631x–OA639x, OB631x–OB639x, OA641x, OA642x, OA647x, O0161x–O0171x, O1641x–O1647x |

*EDI* Electronic Data Interchange, *ICD* International Classification of Diseases
