# Supplementary material for: Cardiovascular outcomes between COVID-19 and non-COVID-19 pneumonia: a nationwide cohort study
Source: BMC Med. 2023 Oct 20;21:394. doi: 10.1186/s12916-023-03106-z (PMC10588072; doi:10.1186/s12916-023-03106-z)
Supplement: Supplementary file 2 — Additional file 2: Table S1. Coding for cardiovascular diseases. Table S2. Comorbidities based on the Charlson Comorbidity Index. Table S3. Types and codes for cardiovascular drug therapies.Table S4. ICD-10-based classification of organ dysfunction. Table S5. Baseline characteristics of participants in the COVID-19 and non-COVID-19 pneumonia groups who died within the first 30 days following the index date. Table S6. Cardiovascular outcomes of participants in the COVID-19 and non-COVID-19 pneumonia groups who died within the first 30 days following the index date. Table S7. Comparison of the risk of cardiovascular outcomes in participants hospitalized for COVID-19 or non-COVID-19 pneumonia. Table S8. Subgroup analyses of the risk of cardiovascular outcomes in participants hospitalized for COVID-19 or non-COVID-19 pneumonia. Table S9. Baseline characteristics of participants in the COVID-19 and non-COVID-19 pneumonia groups, including patients with preexisting cardiovascular disease. Table S10. Comparison of the risk of cardiovascular outcomes in participants hospitalized for COVID-19 or non-COVID-19 pneumonia, in cohorts including patients with preexisting cardiovascular disease. Table S11. Subgroup analyses of the risk of cardiovascular outcomes in participants hospitalized for COVID-19 or non-COVID-19 pneumonia, in cohorts including patients with preexisting cardiovascular disease. Table S12. Baseline characteristics of participants in the COVID-19 and non-COVID-19 pneumonia groups, including only patients with body mass index and smoking status data. Table S13. Subgroup analyses of the risk of cardiovascular outcomes in participants hospitalized for COVID-19 or non-COVID-19 pneumonia, in cohorts that included only patients with body mass index and smoking status data. Table S14. Comparison of the risk of cerebrovascular outcomes, identified using only the International Classification of Diseases, 10th Revision codes, in participants hospitalized for COVID-19 or n [file 12916_2023_3106_MOESM2_ESM.docx]

**Table S1** Coding for cardiovascular diseases

| Variable | ICD-10 or EDI codes |
| --- | --- |
| Stroke |  |
| Ischemic | I63 |
| Hemorrhagic | I60, I61, I62 |
| Ischemic or hemorrhagic | I64 |
| TIA | G45 |
| Atrial fibrillation | I480, I481, I482, I4890 |
| Atrial flutter | I483, I484, I4891 |
| Ventricular arrhythmias | I470, I472, I490, I493 |
| Acute pericarditis | I30 |
| Acute myocarditis | I40 |
| Myocardial infarction | I21, I22, I252 |
| Congestive heart failure | I099, I110, I130, I132, I255, I420, I425, I426, I427, I428, I429, I43, I50 |
| Cardiac arrest | I46, M5873, M5874, M5875, M5876, M5877, M5880 |
| Pulmonary embolism | I26 |
| Deep vein thrombosis | I802 |

*EDI* Electronic Data Interchange, *ICD* International Classification of Diseases, *TIA* transient ischemic attack

**Table S2** Comorbidities based on the Charlson Comorbidity Index^a^

| Variable | ICD-10 codes |
| --- | --- |
| **Charlson Comorbidity Index** |  |
| Myocardial infarction | I21, I22, I252 |
| Congestive heart failure | I099, I110, I130, I132, I255, I420, I425, I426, I427, I428, I429, I43, I50, P290 |
| Peripheral vascular disease | I70, I71, I731, I738, I739, I771, I790, I792, K551, K558, K559, Z958, Z959 |
| Cerebrovascular disease | G45, I60, I61, I62, I63, I64 |
| Dementia | F00, F01, F02, F03, G30, F051, G311 |
| Chronic pulmonary disease | I278, I279, J40, J41, J42, J43, J44, J45, J46, J47, J60, J61, J62, J63, J64, J65, J66, J67, J684, J701, J703 |
| Rheumatic disease | M05, M06, M315, M32, M33, M34, M351, M353, M360 |
| Peptic ulcer disease | K25, K26, K27, K28 |
| Mild liver disease | B18, K700, K701, K702, K703, K709, K713, K714, K715, K717, K73, K74, K760, K762, K763, K764, K768, K769, Z944 |
| Moderate or severe liver disease | I850, I859, I864, I982, K704, K711, K721, K729, K765, K766, K767 |
| Diabetes without complications | E100, E101, E106, E108, E109, E110, E111, E116, E118, E119, E120, E121, E126, E128, E129, E130, E131, E136, E138, E139, E140, E141, E146, E148, E149 |
| Diabetes with complications | E102, E103, E104, E105, E107, E112, E113, E114, E115, E117, E122, E123, E124, E125, E127, E132, E133, E134, E135, E137, E142, E143, E144, E145, E147 |
| Paraplegia and hemiplegia | G041, G114, G800, G81, G82, G830, G831, G832, G833, G834, G839 |
| Renal disease | I120, I131, N030, N031, N032, N033, N034, N035, N036, N037, N038, N039, N050, N051, N052, N053, N054, N055, N056, N057, N058, N059, N18, N19, N250, Z490, Z491, Z492, Z940, Z992 |
| Any malignancy | C00, C01, C02, C03, C04, C05, C06, C07, C08, C09, C10, C11, C12, C13, C14, C15, C16, C17, C18, C19, C20, C21, C22, C23, C24, C25, C26, C30, C31, C32, C33, C34, C37, C38, C39, C40, C41, C43, C45, C46, C47, C48, C49, C50, C51, C52, C53, C54, C55, C56, C57, C58, C60, C61, C62, C63, C64, C65, C66, C67, C68, C69, C70, C71, C72, C73, C74, C75, C76, C81, C82, C83, C84, C85, C88, C90, C91, C92, C93, C94, C95, C96, C97 |
| Metastatic solid tumor | C77, C78, C79, C80 |
| AIDS/HIV | B20, B21, B22, B24 |
| **Hypertension, uncomplicated** | I10 |
| **Hypertension, complicated** | I11, I12, I13, I15 |
| **Dyslipidemia** | E78 |

*AIDS* acquired immune deficiency syndrome, *HIV* human immunodeficiency virus, *ICD* International Classification of Diseases

^a^ Hypertension and dyslipidemia are not included in the Charlson Comorbidity Index and were identified separately using the ICD-10 codes

**Table S3** Types and codes for cardiovascular drug therapies

| Variable | ATC codes | HIRA charge codes |
| --- | --- | --- |
| **ACE inhibitor** |  |  |
| Alacepril | NA | 104201ATB, 104202ATB |
| Captopril | C09AA01 | 122901ATB, 122901ATB, 122903ATB, 262200ATB, 262300ATB |
| Cilazapril | C09AA08 | 133001ATB, 133002ATB, 133003ATB |
| Enalapril maleate | C09AA02 | 151601ATB, 151603ATB, 440300ATB |
| Fosinopril sodium | C09AA09 | 163501ATB, 163502ATB |
| Imidapril hydrochloride | C09AA16 | 173401ATB, 173402ATB |
| Lisinopril | C09AA03 | 184501ATB |
| Perindopril tertbutylamine/arginine | C09AA04 | 211301ATB, 211302ATB, 501601ATB, 501602ATB |
| Quinapril hydrocholoride | C09AA06 | 221901ATB |
| Ramipril | C09AA05 | 222401ATB, 222402ATB, 222404ATB |
| Temocapril hydrocholoride | C09AA14 | 235002ATB |
| Zofenopril calcium | C09AA15 | 510401ATB, 510402ATB, 510403ATB |
| **ARB** |  |  |
| Candesartan cilexetil | C09CA06 | 122601ATB, 122602ATB, 122603ATB, 423700ATB, 661800ATB, 661900ATB, 662000ATB, 662100ATB, 673700ATB |
| Irbesartan | C09CA04 | 177301ATB, 177303ATB |
| Losartan potassium | C09CA01 | 185701ATB, 185702ATB |
| Valsartan | C09CA03 | 247101ATB, 247102ATB, 247103ATB, 247104ATB |
| Telmisartan | C09CA07 | 378801ATB, 378802ATB |
| Eprosartan mesylate | C09CA02 | 429201ATB, 460500ATB |
| Olmesartan medoxomil/cilexetil | C09CA08 | 468501ATB, 468502ATB, 468503ATB, 526300ATB, 526400ATB, 526500ATB, 526900ATB, 547800ATB, 547900ATB, 548000ATB, 631300ATB, 632800ATB, 632900ATB, 633000ATB, 644100ATB, 644200ATB, 653200ATB, 520901ATB, 520902ATB |
| Fimasartan potassium trihydrate | C09CA10 | 515201ATB, 515202ATB, 515203ATB, 522000ATB, 526800ATB, 654600ATB, 654700ATB, 654800ATB, 654900ATB, 655000ATB |
| Sacubitril/valsartan sodium hydrate | C09DX04 | 651401ATB, 651402ATB, 651403ATB |
| Azilsartan medoxomil potassium | C09CA09 | 662401ATB, 662402ATB, 662403ATB, 673500ATB, 673600ATB |
| **Beta-blocker** |  |  |
| Amosulalol hydrocholoride | NA | 107901ATB, 107902ATB |
| Arotinolol hydrocholoride | NA | 110201ATB, 110202ATB |
| Atenolol | C07AB03 | 111402ATB, 111403ATB, 262100ATB, 460200ATB |
| Betaxolol hydrocholoride | C07AB05 | 116801ATB, 116803ATB |
| Bevantolol hydrocholoride | C07AB06 | 117001ATB, 117002ATB |
| Bisoprolol fumarate | C07AB07 | 117901ATB, 117902ATB, 117903ATB, 117904ATB, 469800ATB, 469900ATB, 470000ATB |
| Carteolol hydrocholoride | C07AA15 | 124801ATB |
| Carvedilol | C07AG02 | 125001ATB, 125002ATB, 125003ATB, 125004ACR, 125005ATB, 125006ACR, 125007ACR, 125008ACR, 662201ATB, 662202ATB |
| Celiprolol hydrocholoride | C07AB08 | 129101ATB |
| Metoprolol tartrate/succinate | C07AB02 | 193802ATB, 194003ATR |
| Nadolol | C07AA12 | 198301ATB |
| Propranolol hydrocholoride | C07AA05 | 219901ATB, 219904ATB |
| Sotalol hydrocholoride | C07AA07 | 230402ATB |
| S-atenolol | C07AB11 | 483101ATB, 483102ATB |
| Nebivolol hydrocholoride | C07AB12 | 489501ATB, 489502ATB, 489503ATB |
| **Calcium-channel blocker** |  |  |
| Amlodipine | C08CA01 | 107601ATB, 107601ATD, 107602ATB, 107602ATD, 472300ATB, 472400ATB, 472500ATB, 492800ATB, 492900ATB, 495800ATB, 500500ATB, 500600ATB, 511500ATB, 511600ATB, 511700ATB, 518900ATB, 519700ATB, 519800ATB, 519900ATB, 520000ATB, 520100ATB, 582200ATB, 582400ATB, 623100ATB, 637400ATB, 637500ATB, 637600ATB, 651900ATB, 652000ATB, 652100ATB, 652700ATB, 652900ATB, 653000ATB, 653100ATB, 663500ATB, 663600ATB, 663700ATB, 663800ATB, 671200ATB, 671300ATB, 671400ATB, 671500ATB, 671600ATB, 671700ATB, 673900ATB, 674000ATB, 674100ATB, 459801ACH, 459801ATB, 459802ACH, 459901ATB, 547500ATB, 547600ATB, 547700ATB, 464601ATB, 522900ATB, 523000ATB, 523100ATB, 470801ATB, 470802ATB, 476201ATB, 523200ATB, 523300ATB, 523400ATB, 629400ATB, 629500ATB, 629600ATB, 479701ATB, 483201ATB, 483202ATB, 521200ATB, 521300ATB, 521400ATB, 522600ATB, 522700ATB, 522800ATB, 644800ATB, 486501ATB, 486502ATB, 495901ATB, 502700ATB, 503000ATB, 513900ATB, 662800ATB, 662900ATB, 663000ATB, 663900ATB, 664000ATB, 664100ATB, 664200ATB, 664300ATB, 664400ATB |
| Barnidipine hydrocholoride | C08CA12 | 114001ACH, 114002ACH, 114003ACH |
| Benidipine hydrocholoride | C08CA15 | 115101ATB, 115102ATB, 115103ATB, 115104ATB |
| Cilnidipine | C08CA14 | 133101ATB, 133102ATB |
| Diltiazem hydrocholoride | C08DB01 | 145703ACR, 145706ATB, 145707ACR, 145707ATR |
| Felodipine | C08CA02 | 157501ATR, 157503ATR, 262400ATR, 447100ATB, 447200ATB |
| Isradipine | C08CA03 | 178902ACR |
| Lacidipine | C08CA09 | 180301ATB, 180302ATB, 180303ATB |
| Lercanidipine hydrocholoride | C08CA13 | 182001ATB, 182002ATB, 522200ATB, 522300ATB, 522400ATB |
| Manidipine hydrocholoride | C08CA11 | 188001ATB, 188002ATB |
| Nicardipine hydrocholoride | C08CA04 | 201002ATB, 201003ACR |
| Nifedipine | C08CA05 | 201401ATB, 201405ATR, 201407ACS, 201409ATR, 528201ATR, 528202ATR |
| Nimodipine | C08CA06 | 201901ATB |
| Verapamil hydrocholoride | C08DA01 | 247603ATR, 247605ATR, 247606ATB, 247607ATB |
| Nisoldipine | C08CA07 | 356201ATB, 356202ATB, 356202ATR, 356203ATR |
| Efonidipine | NA | 441201ATB, 441202ATB |
| **Diuretic** |  |  |
| Azosemide | NA | 112901ATB |
| Furosemide | C03CA01 | 163801ATB |
| Hydrochlorothiazide | C03AA03 | 170801ATB, 262500ATB, 262600ATB, 262700ATB, 356400ATB, 378900ATB, 385700ATB, 385800ATB, 442600ATB, 443200ATB, 443300ATB, 448600ATB, 448700ATB, 486900ATB, 499200ATB, 499300ATB, 502600ATB, 513600ATB |
| Indapamide | C03BA11 | 174401ATR, 174403ATB, 556200ATB |
| Amiloride hydrocholoride | C03DB01 | 106901ATB |
| Spironolactone | C03DA01 | 231101ATB, 231102ATB |
| Torasemide | C03CA04 | 242001ATB, 242002ATB, 242003ATB, 242004ATB |
| Xipamide | C03BA10 | 249401ATB |
| Chlorthalidone | C03BA04 | 451302ATB |
| Tolvaptan | C03XA01 | 616501ATB, 616502ATB |
| **Statin** |  |  |
| Atorvastatin calcium | C10AA05 | 111501ATB, 111502ATB, 111503ATB, 111504ATB, 524000ATB, 524100ATB, 527000ATB, 527100ATB, 633800ATB, 633900ATB, 634800ATB |
| Fluvastatin | C10AA04 | 162401ACH, 162402ACH, 162403ATR |
| Pravastatin sodium | C10AA03 | 216601ATB, 216602ATB, 216603ATB, 216604ATB |
| Simvastatin | C10AA01 | 227801ATB, 227801ATR, 227802ATB |
| Rosuvastatin calcium | C10AA07 | 454001ATB, 454002ATB, 454003ATB, 525000ATB, 525100ATB, 525200ATB, 525300ATB, 629700ATB, 629800ATB, 629900ATB, 630000ATB, 630100ATB, 630200ATB, 631600ATB, 631700ATB |
| Pitavastatin calcium | C10AA08 | 470901ATB, 470902ATB, 470903ATB, 634900ATB, 635000ATB, 635100ATB, 635200ATB |
| **Insulin** |  |  |
| Human insulin | A10AE01 | 170130BIJ, 170131BIJ, 170430BIJ, 170431BIJ |
| Insulin lispro | A10AD04 | 175330BIJ, 175331BIJ, 175332BIJ, 175333BIJ |
| Insulin aspart | A10AD05 | 441330BIJ, 441331BIJ, 441332BIJ, 441333BIJ, 441334BIJ, 626700BIJ |
| Insulin glargine | A10AE04 | 461830BIJ, 461831BIJ, 461832BIJ, 666700BIJ, 667000BIJ |
| Insulin glulisine | A10AB06 | 484930BIJ, 484931BIJ |
| Insulin detemir | A10AE05 | 488730BIJ |
| Insulin degludec | A10AE06 | 626830BIJ, 626831BIJ |
| **Other hypoglycemic agents** |  |  |
| Sulfonylureas | A10BB | 165402ATB, 165602ATB, 165603ATR, 165604ATR, 165701ATB, 165702ATB, 165703ATB, 165704ATB, 165801ATB, 443400ATB, 443500ATB, 471900ATB, 474200ATB, 474300ATB, 474300ATR, 497200ATB, 498600ATB, 525500ATB, 525600ATB |
| Meglitinides | A10BX | 379501ATB, 379502ATB, 379503ATB, 430201ATB, 430202ATB, 430203ATB, 486101ATB |
| Biguanides | A10BA | 191501ATB, 191502ATB, 191502ATR, 191503ATB, 191504ATB, 191504ATR, 191505ATR, 498100ATB, 502300ATB, 502300ATR, 502900ATB, 507000ATB, 507100ATB, 513700ATB, 513700ATR, 518500ATR, 518600ATR, 518800ATB, 519600ATB, 523600ATB, 523700ATB, 524700ATR, 631900ATB, 632100ATB, 637200ATB, 641800ATR, 641900ATR, 642000ATR, 644900ATB, 672500ATR, 672600ATR, 672700ATR, 672800ATR, 672900ATR, 673000ATR |
| Thiazolidinediones | A10BG | 431901ATB, 431902ATB, 525901ATB, 653800ATR, 653900ATR, 654000ATR, 655700ATR |
| α-Glucosidase inhibitors | A10BF | 100601ATB, 100602ATB, 249001ATB, 249001ATD, 249002ATB, 249002ATD, 406201ATB |
| DPP-4 inhibitors | A10BH | 500801ATB, 501101ATB, 501102ATB, 501103ATB, 520500ATB, 520600ATB, 520700ATB, 523800ATR, 613301ATB, 613302ATB, 616401ATB, 619101ATB, 624201ATB, 624202ATB, 624203ATB, 627301ATB, 630300ATB, 630400ATB, 630500ATB, 630600ATB, 632000ATR, 635600ATB, 635700ATB, 639601ATB, 645000ATR, 648400ATB, 648500ATB, 648600ATB, 649900ATR, 650000ATR, 650100ATR, 654100ATR, 664600ATB, 664700ATB, 664800ATB |
| SGLT2 inhibitors | A10BK | 527301ATB, 527302ATB, 628201ATB, 628202ATB, 636101ATB, 639800ATR, 641400ATR, 649000ATB, 649100ATB, 649200ATB, 649300ATB, 649400ATB, 649500ATB, 674301ATB, 674302ATB |
| GLP-1 analogues | A10BJ | 512130BIJ, 512131BIJ, 626630BIJ, 626631BIJ, 639701BIJ, 639702BIJ |
| **Antiplatelet agents** |  |  |
| Aspirin | B01AC06 | 110701ATB, 110702ATB, 110801ATB, 111001ACE, 111001ATB, 111001ATE, 111002ATE, 111003ACE, 111003ATE, 489700ACR, 517900ACE, 517900ATE, 667500ACE |
| Cilostazol | B01AC23 | 133201ACR, 133201ATB, 133202ATB, 506100ATB |
| Clopidogrel | B01AC04 | 136901ATB, 492501ATB |
| Saprogrelate hydrochloride | NA | 226101ATB |
| Ticlopidine hydrochloride | B01AC05 | 239201ATB, 239202ATB |
| Tirofiban hydrocholoride monohydrate | B01AC17 | 240230BIJ |
| Triflusal | B01AC18 | 244101ACE |
| Prasugrel hydrochloride | B01AC22 | 597301ATB, 597302ATB |
| Ticagrelor | B01AC24 | 615901ATB, 615902ATB |

*ACE* angiotensin-converting enzyme, *ARB* angiotensin II receptor blocker, *ATC* Anatomic Therapeutic Chemical, *DPP-4* dipeptidyl peptidase-4, *GLP-1* glucagon-like peptide-1, *HIRA* Health Insurance Review and Assessment Service, *SGLT2* sodium-glucose co-transporter-2

**Table S4** ICD-10-based classification of organ dysfunction

| Variable | Codes |
| --- | --- |
| **Cardiovascular** |  |
| Septic shock | R572 |
| Hypotension | I95 |
| Other hypotension | I958 |
| Hypotension, unspecified | I959 |
| Shock, NEC | R57 |
| Other shock | R578 |
| Shock, unspecified | R579 |
| Shock (endotoxic, hypovolemic) during or following a procedure | T811 |
| Use of a vasopressor (norepinephrine, epinephrine, vasopressin, dopamine) |  |
| **Respiratory** |  |
| Adult respiratory distress syndrome | J80 |
| Pulmonary edema | J81 |
| Respiratory failure, NEC | J96 |
| Acute respiratory failure | J960 |
| Respiratory failure, unspecified | J969 |
| Hypoxemia | R0902 |
| Cyanosis | R230 |
| Dependence on respirator | Z991 |
| Conventional oxygen therapy, high-flow nasal cannula, or mechanical ventilation |  |
| **Neurologic** |  |
| Delirium not induced by alcohol and other psychoactive substances | F05 |
| Other mental disorders due to brain damage and dysfunction and due to physical disease | F06 |
| Organic psychosis NOS | F09 |
| Anoxic brain damage, NEC | G931 |
| Encephalopathy, unspecified | G934 |
| Metabolic encephalopathy | G9380 |
| Somnolence, stupor, and coma | R40 |
| Somnolence | R400 |
| Stupor | R401 |
| Disorientation, unspecified | R410 |
| **Hematologic** |  |
| Disseminated intravascular coagulation (defibrination syndrome) | D65 |
| Other coagulation defects | D68 |
| Other specified coagulation defects | D688 |
| Coagulation defect, unspecified | D689 |
| Purpura and other hemorrhagic conditions | D69 |
| Secondary thrombocytopenia | D695 |
| Thrombocytopenia, unspecified | D696 |
| Spontaneous ecchymoses | R233 |
| Abnormal coagulation lab | R791 |
| **Hepatic** |  |
| Hepatic failure, NEC | K72 |
| Central hemorrhagic necrosis of liver | K762 |
| Infarction of liver | K763 |
| Unspecified jaundice | R17 |
| **Renal** |  |
| Acute renal failure | N17 |
| Unspecified renal failure | N19 |
| Postprocedural renal failure | N990 |
| Anuria and oliguria | R34 |
| Abnormal results of kidney function studies | R944 |
| Dependence on renal dialysis | Z992 |
| Renal replacement therapy |  |
| **Metabolic** |  |
| Acidosis | E872 |

*ICD* International Classification of Diseases, *NEC* not elsewhere classified, *NOS* not otherwise specified

**Table S5** Baseline characteristics of participants in the COVID-19 and non-COVID-19 pneumonia groups who died within the first 30 days following the index date

| Characteristics | COVID-19  (n = 694) | Non-COVID-19 pneumonia  (n = 1464) | SMD |
| --- | --- | --- | --- |
| Age, mean (SD), y | 79.2 (12.4) | 76.6 (13.4) | 0.08 |
| Sex, No. (%) |  |  | 0.06 |
| Male | 334 (48.1) | 832 (56.8) |  |
| Female | 360 (51.9) | 632 (43.2) |  |
| Comorbidities, No. (%) |  |  |  |
| Diabetes | 275 (39.6) | 514 (35.1) | 0.09 |
| Hypertension | 441 (63.5) | 794 (54.2) | 0.19 |
| Dyslipidemia | 302 (43.5) | 497 (34.0) | 0.20 |
| Peripheral vascular disease | 111 (16.0) | 235 (16.1) | –0.002 |
| Chronic pulmonary disease | 197 (28.4) | 710 (48.5) | –0.42 |
| Chronic liver disease | 157 (22.6) | 330 (22.5) | 0.002 |
| Chronic kidney disease | 24 (3.5) | 40 (2.7) | 0.04 |
| Malignancy | 79 (11.4) | 360 (24.6) | –0.35 |
| Charlson Comorbidity Index, mean (SD) | 3.0 (1.7) | 2.6 (1.7) | 0.18 |
| Income level, No. (%) |  |  | 0.22 |
| Q1 (lowest) | 184 (26.5) | 368 (25.1) |  |
| Q2 | 184 (26.5) | 392 (26.8) |  |
| Q3 | 159 (22.9) | 431 (29.4) |  |
| Q4 (highest) | 166 (23.9) | 263 (18.0) |  |
| Missing | 1 (0.1) | 10 (0.7) |  |
| Hospital size, No. (%) |  |  | 0.26 |
| <500 beds | 416 (59.9) | 878 (60.0) |  |
| ≥500 beds | 265 (38.2) | 487 (33.3) |  |
| Missing | 13 (1.9) | 99 (6.8) |  |
| Cardiovascular drug therapy, No. (%) |  |  |  |
| ACE inhibitor | 1 (0.1) | 2 (0.1) | 0.002 |
| ARB | 40 (5.8) | 36 (2.5) | 0.17 |
| Beta-blocker | 19 (2.7) | 26 (1.8) | 0.06 |
| Calcium-channel blocker | 74 (10.7) | 96 (6.6) | 0.15 |
| Diuretic | 90 (13.0) | 167 (11.4) | 0.05 |
| Statin | 33 (4.8) | 28 (1.9) | 0.16 |
| Insulin | 64 (9.2) | 69 (4.7) | 0.18 |
| Other hypoglycemic agents | 46 (6.6) | 52 (3.6) | 0.14 |
| Antiplatelet agents | 44 (6.3) | 44 (3.0) | 0.16 |
| Organ dysfunction, No. (%) |  |  |  |
| Cardiovascular | 444 (64.0) | 716 (48.9) | 0.31 |
| Respiratory | 656 (94.5) | 1319 (90.1) | 0.17 |
| Neurologic | 17 (2.5) | 74 (5.1) | –0.14 |
| Hematologic | 47 (6.8) | 40 (2.7) | 0.19 |
| Hepatic | 5 (0.7) | 17 (1.2) | –0.05 |
| Renal | 108 (15.6) | 218 (14.9) | 0.02 |
| Metabolic | 20 (2.9) | 15 (1.0) | 0.13 |
| Vasopressor use, No. (%) | 441 (63.5) | 703 (48.0) | 0.32 |
| Oxygen therapy, No. (%) |  |  |  |
| No oxygen | 39 (5.6) | 151 (10.3) | –0.17 |
| Supplemental oxygen | 497 (71.6) | 1223 (83.5) | –0.29 |
| High-flow nasal cannula | 411 (59.2) | 353 (24.1) | 0.76 |
| Mechanical ventilation | 363 (52.3) | 405 (27.7) | 0.52 |
| Renal replacement therapy, No. (%) | 55 (7.9) | 110 (7.5) | 0.02 |
| ECMO, No. (%) | 20 (2.9) | 4 (0.3) | 0.21 |

*ACE* angiotensin-converting enzyme, *ARB* angiotensin II receptor blocker, *COVID-19* coronavirus disease 2019, *ECMO* extracorporeal membrane oxygenation, *SMD* standardized mean difference

**Table S6** Cardiovascular outcomes of participants in the COVID-19 and non-COVID-19 pneumonia groups who died within the first 30 days following the index date

| Outcomes | COVID-19  (n = 694) | Non-COVID-19 pneumonia  (n = 1464) | *P* value |
| --- | --- | --- | --- |
| Stroke, No. (%) | 4 (0.6) | 10 (0.7) | 0.77 |
| Ischemic | 3 (0.4) | 9 (0.6) | 0.76 |
| Hemorrhagic | 1 (0.1) | 2 (0.1) | >0.99 |
| TIA, No. (%) | 0 | 0 |  |
| Atrial fibrillation, No. (%) | 18 (2.6) | 20 (1.4) | 0.04 |
| Atrial flutter, No. (%) | 0 | 0 |  |
| Ventricular arrhythmias, No. (%) | 3 (0.4) | 7 (0.5) | >0.99 |
| Acute pericarditis, No. (%) | 0 | 0 |  |
| Acute myocarditis, No. (%) | 0 | 0 |  |
| Myocardial infarction, No. (%) | 14 (2.0) | 39 (2.7) | 0.36 |
| Congestive heart failure, No. (%) | 56 (8.1) | 151 (10.3) | 0.10 |
| Cardiac arrest, No. (%) | 49 (7.1) | 81 (5.5) | 0.16 |
| Pulmonary embolism, No. (%) | 10 (1.4) | 24 (1.6) | 0.73 |
| Deep vein thrombosis, No. (%) | 24 (3.5) | 4 (0.3) | <0.001 |

*COVID-19* coronavirus disease 2019, *TIA* transient ischemic attack

**Table S7** Comparison of the risk of cardiovascular outcomes in participants hospitalized for COVID-19 or non-COVID-19 pneumonia

| Outcomes | No. of events | | Incidence per 1000000 person-days | | Hazard ratio  (95% CI) | *P* value |
| --- | --- | --- | --- | --- | --- | --- |
|  | COVID-19  (n = 125810) | Non-COVID-19 pneumonia  (n = 28492) | COVID-19 | Non-COVID-19 pneumonia |  |  |
| **Primary outcome** |  |  |  |  |  |  |
| Major adverse cardiovascular event^a^ | 386 (0.3) | 161 (0.6) | 14.8 | 21.2 | 0.84 (0.69–1.03) | 0.10 |
| **Secondary outcomes** |  |  |  |  |  |  |
| All-cause mortality | 793 (0.6) | 757 (2.7) | 30.4 | 99.8 | 0.25 (0.23–0.28) | <0.001 |
| Stroke | 111 (0.1) | 43 (0.2) | 4.3 | 5.6 | 0.85 (0.58–1.24) | 0.39 |
| Ischemic | 79 (0.1) | 30 (0.1) | 3.0 | 3.9 | 0.81 (0.52–1.26) | 0.34 |
| Hemorrhagic | 31 (0.02) | 14 (0.1) | 1.2 | 1.9 | 0.75 (0.37–1.49) | 0.41 |
| TIA | 34 (0.03) | 8 (0.03) | 1.3 | 1.0 | 1.54 (0.68–3.50) | 0.30 |
| Atrial fibrillation | 369 (0.3) | 118 (0.4) | 14.1 | 15.5 | 0.91 (0.73–1.14) | 0.42 |
| Atrial flutter | 25 (0.02) | 2 (0.01) | 0.9 | 0.2 | 3.80 (0.90–16.09) | 0.07 |
| Ventricular arrhythmias | 103 (0.1) | 41 (0.1) | 3.9 | 5.4 | 0.96 (0.66–1.38) | 0.81 |
| Acute pericarditis | 21 (0.02) | 3 (0.01) | 0.8 | 0.4 | 3.36 (0.79–14.36) | 0.10 |
| Acute myocarditis | 76 (0.1) | 6 (0.02) | 2.9 | 0.8 | 6.47 (2.53–16.52) | <0.001 |
| Myocardial infarction | 280 (0.2) | 119 (0.4) | 10.7 | 15.6 | 0.85 (0.67–1.07) | 0.17 |
| Congestive heart failure | 1235 (1.0) | 659 (2.3) | 47.3 | 86.8 | 0.66 (0.60–0.73) | <0.001 |
| Cardiac arrest | 103 (0.1) | 101 (0.4) | 3.9 | 13.3 | 0.38 (0.27–0.53) | <0.001 |
| Pulmonary embolism | 346 (0.3) | 103 (0.4) | 13.2 | 13.6 | 1.07 (0.84–1.35) | 0.60 |
| Deep vein thrombosis | 237 (0.2) | 45 (0.2) | 9.1 | 5.9 | 1.97 (1.38–2.80) | <0.001 |

The numbers of the cumulative incidence and the outcome-specific incidence rates are shown. The hazard ratios and 95% CIs have been estimated in the inverse probability of treatment-weighted COVID-19 and non-COVID-19 pneumonia groups

*CI* confidence interval, *COVID-19* coronavirus disease 2019, *TIA* transient ischemic attack

^a^ A composite of myocardial infarction and stroke

**Table S8** Subgroup analyses of the risk of cardiovascular outcomes in participants hospitalized for COVID-19 or non-COVID-19 pneumonia

| Subgroup | Hazard ratio (95% CI) - COVID-19 vs Non-COVID-19 pneumonia | | | | | | | | |
| --- | --- | --- | --- | --- | --- | --- | --- | --- | --- |
|  | MACE^a^ | All-cause mortality | Cerebrovascular disease | Dysrhythmia | Inflammatory heart disease | Myocardial infarction | Congestive heart failure | Cardiac arrest | Thrombotic disease |
| **Age, years** | | | | | | | | | |
| 18–39 | 7.30  (3.29–16.21) | 0.12  (0.05–0.27) | 3.92  (0.62–24.70) | 1.88  (1.04–3.42) | 11.33  (2.97–43.20) | 6.78  (3.03–15.15) | 1.95  (1.37–2.77) | 0.10  (0.03–0.34) | 8.26  (4.06–16.83) |
| 40–64 | 0.75  (0.55–1.02) | 0.18  (0.14–0.22) | 1.21  (0.64–2.27) | 0.84  (0.60–1.18) | 3.65  (1.10–12.14) | 0.68  (0.48–0.97) | 0.61  (0.52–0.71) | 0.29  (0.15–0.56) | 1.05  (0.78–1.42) |
| ≥65 | 0.58  (0.45–0.75) | 0.28  (0.24–0.31) | 0.58  (0.38–0.89) | 0.76  (0.60–0.97) | 0.89  (0.17–4.58) | 0.56  (0.41–0.77) | 0.51  (0.44–0.58) | 0.43  (0.28–0.65) | 1.15  (0.84–1.58) |
| **Sex** | | | | | | | | | |
| Male | 0.77  (0.59–1.01) | 0.21  (0.18–0.24) | 0.75  (0.46–1.22) | 0.68  (0.53–0.87) | 6.53  (1.68–25.45) | 0.80  (0.59–1.08) | 0.37  (0.30–0.45) | 0.22  (0.15–0.33) | 1.02  (0.77–1.36) |
| Female | 0.95  (0.70–1.29) | 0.36  (0.30–0.43) | 1.09  (0.64–1.85) | 1.27  (0.95–1.70) | 4.45  (1.68–11.77) | 0.93  (0.65–1.33) | 0.52  (0.45–0.61) | 0.98  (0.51–1.91) | 2.17  (1.59–2.95) |
| **Diabetes** | | | | | | | | | |
| No | 0.89  (0.70–1.14) | 0.21  (0.18–0.24) | 0.76  (0.49–1.18) | 0.78  (0.62–0.98) | 4.73  (2.11–10.60) | 0.99  (0.74–1.33) | 0.69  (0.61–0.78) | 0.27  (0.18–0.41) | 1.57  (1.23–2.01) |
| Yes | 0.76  (0.55–1.06) | 0.36  (0.30–0.42) | 1.23  (0.67–2.28) | 1.27  (0.89–1.81) | 18.57  (0.35–990.53) | 0.61  (0.41–0.90) | 0.61  (0.51–0.73) | 0.62  (0.35–1.12) | 1.27  (0.87–1.84) |
| **Hypertension** | | | | | | | | | |
| No | 1.07  (0.80–1.43) | 0.19  (0.16–0.22) | 0.84  (0.50–1.41) | 0.79  (0.61–1.02) | 4.79  (2.10–10.92) | 1.17  (0.83–1.63) | 0.81  (0.69–0.94) | 0.29  (0.19–0.45) | 1.74  (1.34–2.27) |
| Yes | 0.63  (0.48–0.83) | 0.32  (0.28–0.38) | 0.91  (0.56–1.47) | 1.02  (0.77–1.34) | 10.39  (0.52–208.00) | 0.55  (0.40–0.77) | 0.53  (0.46–0.61) | 0.51  (0.30–0.87) | 1.08  (0.78–1.49) |
| **Dyslipidemia** | | | | | | | | | |
| No | 0.85  (0.65–1.13) | 0.28  (0.24–0.32) | 0.70  (0.43–1.14) | 0.70  (0.55–0.89) | 3.61  (1.58–8.26) | 0.92  (0.67–1.26) | 0.63  (0.55–0.73) | 0.21  (0.14–0.33) | 1.49  (1.14–1.95) |
| Yes | 0.86  (0.64–1.15) | 0.30  (0.26–0.36) | 1.22  (0.72–2.05) | 1.28  (0.95–1.72) | 38.52  (1.89–787.06) | 0.79  (0.56–1.11) | 0.72  (0.62–0.83) | 0.74  (0.43–1.27) | 1.49  (1.07–2.07) |
| **Charlson Comorbidity Index** | | | | | | | | | |
| <3 | 0.94  (0.72–1.23) | 0.22  (0.19–0.25) | 0.78  (0.47–1.30) | 0.78  (0.62–0.98) | 5.16  (2.25–11.86) | 1.00  (0.74–1.35) | 0.67  (0.59–0.77) | 0.26  (0.17–0.40) | 1.72  (1.32–2.23) |
| ≥3 | 0.75  (0.56–1.00) | 0.31  (0.27–0.36) | 1.09  (0.66–1.78) | 1.23  (0.89–1.70) | 4.53  (0.37–55.87) | 0.64  (0.44–0.93) | 0.66  (0.57–0.78) | 0.63  (0.36–1.08) | 1.14  (0.82–1.59) |
| **No. of organ dysfunctions** | | | | | | | | | |
| <3 | 0.85  (0.70–1.04) | 0.26  (0.23–0.29) | 0.89  (0.62–1.27) | 0.87  (0.72–1.05) | 5.13  (2.32–11.33) | 0.86  (0.68–1.09) | 0.67  (0.60–0.74) | 0.40  (0.29–0.57) | 1.48  (1.20–1.82) |
| ≥3 | 0.37  (0.08–1.70) | 1.30  (0.76–2.23) | N/A | 2.84  (0.60–13.46) | N/A | 0.37  (0.08–1.69) | 0.26  (0.12–0.56) | 0.07  (0.02–0.38) | 3.79  (0.16–89.50) |
| **Illness severity** | | | | | | | | | |
| Mild to moderate | 0.85  (0.69–1.03) | 0.23  (0.21–0.26) | 0.89  (0.63–1.28) | 0.89  (0.73–1.07) | 5.13  (2.32–11.32) | 0.85  (0.67–1.07) | 0.66  (0.60–0.73) | 0.37  (0.27–0.52) | 1.49  (1.21–1.84) |
| Severe to critical | 0.45  (0.25–0.81) | 0.63  (0.52–0.76) | 0.68  (0.21–2.15) | 1.61  (0.94–2.77) | 0.36  (0.07–1.82) | 0.38  (0.19–0.76) | 0.51  (0.38–0.68) | 0.20  (0.12–0.34) | 1.29  (0.75–2.21) |

The hazard ratios and 95% CIs have been estimated in the inverse probability of treatment-weighted COVID-19 (n = 125810) and non-COVID-19 pneumonia (n = 28492) groups

*CI* confidence interval, *COVID-19* coronavirus disease 2019, *MACE* major adverse cardiovascular event, *N/A* not applicable

^a^ A composite of myocardial infarction and stroke

**Table S9** Baseline characteristics of participants in the COVID-19 and non-COVID-19 pneumonia groups, including patients with preexisting cardiovascular disease

| Characteristics | Before weighting | | | After weighting | | |
| --- | --- | --- | --- | --- | --- | --- |
|  | COVID-19  (n = 140273) | Non-COVID-19 pneumonia  (n = 41576) | SMD | COVID-19  (n = 133376) | Non-COVID-19 pneumonia  (n = 37629) | SMD |
| Age, mean (SD), y | 45.2 (16.3) | 63.3 (19.7) | 1.01 | 48.3 (17.6) | 50.9 (19.6) | 0.03 |
| Sex, No. (%) |  |  | 0.54 |  |  | 0.02 |
| Male | 72593 (51.8) | 19065 (45.9) |  | 65703 (49.3) | 17651 (46.9) |  |
| Female | 67680 (48.2) | 22511 (54.1) |  | 65672 (50.7) | 19978 (53.1) |  |
| Comorbidities, No. (%) |  |  |  |  |  |  |
| Diabetes | 18960 (13.5) | 12494 (30.1) | –0.41 | 22730 (17.0) | 6941 (18.5) | –0.04 |
| Hypertension | 27575 (19.7) | 19277 (46.4) | –0.59 | 33282 (25.0) | 9836 (26.1) | –0.03 |
| Dyslipidemia | 36316 (25.9) | 18394 (44.2) | –0.39 | 39925 (29.9) | 12304 (32.7) | –0.06 |
| Myocardial infarction | 742 (0.5) | 967 (2.3) | –0.15 | 1144 (0.9) | 351 (0.9) | –0.01 |
| Congestive heart failure | 2982 (2.1) | 5137 (12.4) | –0.40 | 5123 (3.8) | 1568 (4.2) | –0.02 |
| Dysrhythmia | 1233 (0.9) | 1662 (4.0) | –0.20 | 1968 (1.5) | 608 (1.6) | –0.01 |
| Cerebrovascular disease^a^ | 2890 (2.1) | 5169 (12.4) | –0.41 | 5009 (3.7) | 1558 (4.1) | –0.02 |
| Thrombotic disease^b^ | 1299 (0.9) | 566 (1.4) | –0.04 | 1219 (1.0) | 387 (1.0) | –0.01 |
| Peripheral vascular disease | 7925 (5.6) | 6880 (16.6) | –0.35 | 10498 (7.9) | 3145 (8.4) | –0.02 |
| Chronic pulmonary disease | 18680 (13.3) | 22381 (53.8) | –0.95 | 28972 (21.7) | 8715 (23.2) | –0.03 |
| Chronic liver disease | 20243 (14.4) | 10228 (24.6) | –0.26 | 22386 (16.8) | 7017 (18.7) | –0.05 |
| Chronic kidney disease | 880 (0.6) | 956 (2.3) | –0.14 | 1162 (0.9) | 405 (1.1) | –0.02 |
| Malignancy | 5479 (3.9) | 4741 (11.4) | –0.28 | 7249 (5.4) | 2297 (6.1) | –0.03 |
| Charlson Comorbidity Index, mean (SD) | 0.9 (1.3) | 2.5 (1.8) | 0.95 | 1.2 (1.5) | 1.4 (1.6) | 0.04 |
| Income level, No. (%) |  |  | 0.17 |  |  | 0.00 |
| Q1 (lowest) | 37561 (26.8) | 11163 (26.9) |  | 36379 (27.3) | 10284 (27.3) |  |
| Q2 | 32887 (23.4) | 10744 (25.8) |  | 32226 (24.2) | 9055 (24.1) |  |
| Q3 | 35895 (25.6) | 10579 (25.4) |  | 34485 (25.9) | 9899 (26.3) |  |
| Q4 (highest) | 33233 (23.7) | 8821 (21.2) |  | 29577 (22.2) | 8196 (21.8) |  |
| Missing | 697 (0.5) | 269 (0.7) |  | 708 (0.5) | 195 (0.5) |  |
| Hospital size, No. (%) |  |  | 0.34 |  |  | 0.09 |
| <500 beds | 102595 (73.1) | 28871 (69.4) |  | 99058 (74.3) | 28269 (75.1) |  |
| ≥500 beds | 36511 (26.0) | 9455 (22.7) |  | 32343 (24.2) | 8685 (23.1) |  |
| Missing | 1167 (0.8) | 3250 (7.8) |  | 1975 (1.5) | 676 (1.8) |  |
| Cardiovascular drug therapy, No. (%) |  |  |  |  |  |  |
| ACE inhibitor | 41 (0.03) | 41 (0.1) | –0.03 | 72 (0.1) | 25 (0.1) | –0.01 |
| ARB | 760 (0.5) | 545 (1.3) | –0.08 | 995 (0.7) | 367 (1.0) | –0.03 |
| Beta-blocker | 957 (0.7) | 534 (1.3) | –0.06 | 1134 (0.9) | 366 (1.0) | –0.01 |
| Calcium-channel blocker | 1338 (1.0) | 1076 (2.6) | –0.12 | 1842 (1.4) | 623 (1.7) | –0.02 |
| Diuretic | 1025 (0.7) | 1130 (2.7) | –0.15 | 1598 (1.2) | 585 (1.6) | –0.03 |
| Statin | 847 (0.6) | 692 (1.7) | –0.10 | 1176 (0.9) | 410 (1.1) | –0.02 |
| Insulin | 812 (0.6) | 629 (1.5) | –0.09 | 1111 (0.8) | 435 (1.2) | –0.03 |
| Other hypoglycemic agents | 992 (0.7) | 788 (1.9) | –0.11 | 1358 (1.0) | 501 (1.3) | –0.03 |
| Antiplatelet agents | 960 (0.7) | 805 (1.9) | –0.11 | 1302 (1.0) | 463 (1.2) | –0.02 |
| Organ dysfunction, No. (%) |  |  |  |  |  |  |
| Cardiovascular | 1881 (1.3) | 2956 (7.1) | –0.29 | 2748 (2.1) | 1029 (2.7) | –0.04 |
| Respiratory | 18328 (13.1) | 13051 (31.4) | –0.45 | 20791 (15.6) | 6346 (16.9) | –0.03 |
| Neurologic | 588 (0.4) | 963 (2.3) | –0.16 | 894 (0.7) | 329 (0.9) | –0.02 |
| Hematologic | 1929 (1.4) | 328 (0.8) | 0.06 | 973 (0.7) | 304 (0.8) | –0.01 |
| Hepatic | 46 (0.03) | 80 (0.2) | –0.05 | 61 (0.1) | 21 (0.1) | –0.01 |
| Renal | 526 (0.4) | 1586 (3.8) | –0.24 | 925 (0.7) | 352 (0.9) | –0.03 |
| Metabolic | 59 (0.04) | 89 (0.2) | –0.05 | 81 (0.1) | 30 (0.1) | –0.01 |
| Vasopressor use, No. (%) | 1753 (1.2) | 2814 (6.8) | –0.28 | 2594 (1.9) | 973 (2.6) | –0.04 |
| Oxygen therapy, No. (%) |  |  |  |  |  |  |
| No oxygen | 121976 (87.0) | 28685 (69.0) | 0.44 | 112647 (84.5) | 31349 (83.3) | 0.03 |
| Supplemental oxygen | 18128 (12.9) | 12826 (30.9) | –0.44 | 20513 (15.4) | 6247 (16.6) | –0.03 |
| High-flow nasal cannula | 3693 (2.6) | 1294 (3.1) | –0.03 | 2879 (2.2) | 1039 (2.8) | –0.04 |
| Mechanical ventilation | 1125 (0.8) | 1245 (3.0) | –0.16 | 1528 (1.1) | 557 (1.5) | –0.03 |
| Renal replacement therapy, No. (%) | 299 (0.2) | 811 (2.0) | –0.17 | 541 (0.4) | 210 (0.6) | –0.02 |
| ECMO, No. (%) | 77 (0.1) | 17 (0.04) | 0.01 | 52 (0.04) | 19 (0.1) | –0.01 |

*ACE* angiotensin-converting enzyme, *ARB* angiotensin II receptor blocker, *COVID-19* coronavirus disease 2019, *ECMO* extracorporeal membrane oxygenation, *SMD* standardized mean difference

^a^ Cerebrovascular disease included stroke and transient ischemic attack

^b^ Thrombotic disease included pulmonary embolism and deep vein thrombosis

**Table S10** Comparison of the risk of cardiovascular outcomes in participants hospitalized for COVID-19 or non-COVID-19 pneumonia, in cohorts including patients with preexisting cardiovascular disease

| Outcomes | No. of events | | Incidence per 1000000 person-days | | Hazard ratio  (95% CI) | *P* value |
| --- | --- | --- | --- | --- | --- | --- |
|  | COVID-19  (n = 133376) | Non-COVID-19 pneumonia  (n = 37629) | COVID-19 | Non-COVID-19 pneumonia |  |  |
| **Primary outcome** |  |  |  |  |  |  |
| Major adverse cardiovascular event^a^ | 1013 (0.8) | 660 (1.8) | 37.7 | 69.4 | 0.46 (0.42–0.51) | <0.001 |
| **Secondary outcomes** |  |  |  |  |  |  |
| All-cause mortality | 1358 (1.0) | 1183 (3.1) | 50.5 | 124.5 | 0.37 (0.34–0.40) | <0.001 |
| Stroke | 209 (0.2) | 258 (0.7) | 7.8 | 27.2 | 0.24 (0.20–0.29) | <0.001 |
| Ischemic | 144 (0.1) | 203 (0.5) | 5.3 | 21.3 | 0.21 (0.17–0.26) | <0.001 |
| Hemorrhagic | 67 (0.1) | 76 (0.2) | 2.5 | 8.0 | 0.27 (0.19–0.37) | <0.001 |
| TIA | 43 (0.03) | 48 (0.1) | 1.6 | 5.1 | 0.27 (0.18–0.41) | <0.001 |
| Atrial fibrillation | 1836 (1.4) | 702 (1.9) | 68.3 | 73.9 | 0.76 (0.69–0.82) | <0.001 |
| Atrial flutter | 77 (0.1) | 22 (0.1) | 2.9 | 2.3 | 1.02 (0.63–1.64) | 0.95 |
| Ventricular arrhythmias | 342 (0.3) | 176 (0.5) | 12.7 | 18.5 | 0.59 (0.50–0.71) | <0.001 |
| Acute pericarditis | 28 (0.02) | 7 (0.02) | 1.0 | 0.8 | 1.34 (0.56–3.17) | 0.51 |
| Acute myocarditis | 79 (0.1) | 13 (0.03) | 2.9 | 1.4 | 1.99 (1.09–3.64) | 0.03 |
| Myocardial infarction | 811 (0.6) | 415 (1.1) | 30.2 | 43.7 | 0.60 (0.53–0.67) | <0.001 |
| Congestive heart failure | 3999 (3.0) | 2162 (5.7) | 148.7 | 227.6 | 0.56 (0.53–0.59) | <0.001 |
| Cardiac arrest | 184 (0.1) | 242 (0.6) | 6.8 | 25.4 | 0.25 (0.20–0.30) | <0.001 |
| Pulmonary embolism | 542 (0.4) | 357 (0.9) | 20.2 | 37.5 | 0.46 (0.40–0.52) | <0.001 |
| Deep vein thrombosis | 380 (0.3) | 191 (0.5) | 14.1 | 20.1 | 0.61 (0.51–0.73) | <0.001 |

The numbers of the cumulative incidence and the outcome-specific incidence rates are shown. The hazard ratios and 95% CIs have been estimated in the inverse probability of treatment-weighted COVID-19 and non-COVID-19 pneumonia groups

*CI* confidence interval, *COVID-19* coronavirus disease 2019, *TIA* transient ischemic attack

^a^ A composite of myocardial infarction and stroke

**Table S11** Subgroup analyses of the risk of cardiovascular outcomes in participants hospitalized for COVID-19 or non-COVID-19 pneumonia, in cohorts including patients with preexisting cardiovascular disease

| Subgroup | Hazard ratio (95% CI) - COVID-19 vs Non-COVID-19 pneumonia | | | | | | | | |
| --- | --- | --- | --- | --- | --- | --- | --- | --- | --- |
|  | MACE^a^ | All-cause mortality | Cerebrovascular disease | Dysrhythmia | Inflammatory heart disease | Myocardial infarction | Congestive heart failure | Cardiac arrest | Thrombotic disease |
| **Age, years** | | | | | | | | | |
| 18–39 | 3.71  (1.98–6.96) | 0.10  (0.05–0.21) | 0.78  (0.32–1.92) | 1.00  (0.67–1.49) | 11.99  (3.46–41.53) | 6.76  (3.25–14.08) | 1.29  (0.99–1.69) | 0.15  (0.05–0.41) | 5.51  (3.10–9.79) |
| 40–64 | 0.45  (0.38–0.54) | 0.18  (0.15–0.22) | 0.27  (0.19–0.37) | 0.60  (0.51–0.70) | 1.67  (0.71–3.94) | 0.56  (0.46–0.68) | 0.54  (0.49–0.59) | 0.20  (0.13–0.29) | 0.52  (0.43–0.64) |
| ≥65 | 0.58  (0.50–0.69) | 0.19  (0.16–0.23) | 0.31  (0.23–0.41) | 0.68  (0.56–0.78) | 3.92  (1.97–7.80) | 0.74  (0.61–0.90) | 0.63  (0.58–0.69) | 0.21  (0.15–0.30) | 0.84  (0.70–1.01) |
| **Sex** | | | | | | | | | |
| Male | 0.45  (0.40–0.51) | 0.26  (0.24–0.29) | 0.21  (0.17–0.27) | 0.63  (0.57–0.70) | 2.96  (1.20–7.31) | 0.58  (0.50–0.68) | 0.53  (0.49–0.57) | 0.20  (0.16–0.26) | 0.42  (0.36–0.49) |
| Female | 0.55  (0.47–0.65) | 0.52  (0.45–0.59) | 0.31  (0.24–0.40) | 0.75  (0.67–0.85) | 2.55  (1.24–5.21) | 0.76  (0.62–0.93) | 0.62  (0.57–0.67) | 0.47  (0.31–0.70) | 0.87  (0.73–1.03) |
| **Diabetes** | | | | | | | | | |
| No | 0.50  (0.43–0.57) | 0.29  (0.26–0.32) | 0.25  (0.20–0.32) | 0.61  (0.55–0.68) | 3.20  (1.70–6.02) | 0.70  (0.59–0.83) | 0.56  (0.52–0.60) | 0.19  (0.14–0.25) | 0.66  (0.57–0.77) |
| Yes | 0.50  (0.43–0.58) | 0.48  (0.42–0.55) | 0.27  (0.21–0.35) | 0.83  (0.74–0.94) | 1.03  (0.27–3.91) | 0.61  (0.51–0.72) | 0.60  (0.55–0.65) | 0.41  (0.30–0.56) | 0.50  (0.42–0.60) |
| **Hypertension** | | | | | | | | | |
| No | 0.58  (0.48–0.70) | 0.23  (0.21–0.27) | 0.30  (0.22–0.42) | 0.56  (0.48–0.66) | 3.50  (1.77–6.91) | 0.78  (0.61–0.98) | 0.57  (0.52–0.64) | 0.19  (0.14–0.27) | 0.82  (0.68–0.98) |
| Yes | 0.46  (0.41–0.52) | 0.45  (0.40–0.50) | 0.24  (0.20–0.29) | 0.75  (0.68–0.82) | 1.08  (0.42–2.79) | 0.59  (0.51–0.68) | 0.57  (0.54–0.61) | 0.33  (0.25–0.43) | 0.46  (0.39–0.53) |
| **Dyslipidemia** | | | | | | | | | |
| No | 0.41  (0.34–0.48) | 0.30  (0.27–0.33) | 0.25  (0.19–0.33) | 0.55  (0.48–0.62) | 2.39  (1.25–4.57) | 0.54  (0.44–0.67) | 0.47  (0.43–0.51) | 0.17  (0.12–0.23) | 0.63  (0.53–0.74) |
| Yes | 0.57  (0.51–0.65) | 0.44  (0.38–0.49) | 0.28  (0.22–0.35) | 0.84  (0.76–0.93) | 3.80  (1.22–11.87) | 0.74  (0.64–0.86) | 0.68  (0.63–0.72) | 0.41  (0.31–0.54) | 0.59  (0.50–0.69) |
| **Charlson Comorbidity Index** | | | | | | | | | |
| <3 | 0.52  (0.45–0.61) | 0.26  (0.23–0.29) | 0.23  (0.17–0.31) | 0.57  (0.50–0.64) | 3.86  (1.94–7.69) | 0.72  (0.60–0.86) | 0.53  (0.49–0.57) | 0.15  (0.11–0.21) | 0.77  (0.65–0.91) |
| ≥3 | 0.50  (0.44–0.57) | 0.45  (0.41–0.51) | 0.30  (0.24–0.37) | 0.88  (0.78–0.98) | 0.72  (0.28–1.89) | 0.62  (0.53–0.73) | 0.65  (0.60–0.70) | 0.42  (0.32–0.56) | 0.48  (0.41–0.57) |
| **No. of organ dysfunctions** | | | | | | | | | |
| <3 | 0.49  (0.45–0.55) | 0.32  (0.29–0.35) | 0.26  (0.22–0.31) | 0.68  (0.62–0.73) | 2.69  (1.53–4.73) | 0.65  (0.57–0.73) | 0.57  (0.54–0.60) | 0.27  (0.22–0.34) | 0.60  (0.53–0.67) |
| ≥3 | 0.43  (0.22–0.84) | 1.26  (0.86–1.83) | 0.05  (0.01–0.49) | 1.24  (0.62–2.50) | NA | 0.56  (0.27–1.18) | 0.43  (0.29–0.65) | 0.25  (0.12–0.51) | 0.60  (0.18–1.97) |
| **Illness severity** | | | | | | | | | |
| Mild to moderate | 0.49  (0.45–0.54) | 0.33  (0.30–0.35) | 0.26  (0.21–0.30) | 0.68  (0.63–0.74) | 2.69  (1.53–4.73) | 0.65  (0.57–0.73) | 0.57  (0.54–0.60) | 0.27  (0.22–0.33) | 0.60  (0.53–0.67) |
| Severe to critical | 0.46  (0.34–0.64) | 0.83  (0.71–0.97) | 0.31  (0.18–0.54) | 0.90  (0.69–1.17) | 0.59  (0.18–1.94) | 0.55  (0.37–0.82) | 0.53  (0.44–0.63) | 0.21  (0.14–0.32) | 0.75  (0.54–1.04) |

The hazard ratios and 95% CIs have been estimated in the inverse probability of treatment-weighted COVID-19 (n = 133376) and non-COVID-19 pneumonia (n = 37629) groups

*CI* confidence interval, *COVID-19* coronavirus disease 2019, *MACE* major adverse cardiovascular event, *N/A* not applicable

^a^ A composite of myocardial infarction and stroke

**Table S12** Baseline characteristics of participants in the COVID-19 and non-COVID-19 pneumonia groups, including only patients with body mass index and smoking status data

| Characteristics | Before weighting | | | After weighting | | |
| --- | --- | --- | --- | --- | --- | --- |
|  | COVID-19  (n = 5409) | Non-COVID-19 pneumonia  (n = 9041) | SMD | COVID-19  (n = 5503) | Non-COVID-19 pneumonia  (n = 9015) | SMD |
| Age, mean (SD), y | 49.7 (14.2) | 56.6 (16.7) | 0.44 | 53.4 (15.4) | 54.5 (16.4) | 0.00 |
| Sex, No. (%) |  |  | 0.17 |  |  | –0.02 |
| Male | 2445 (45.2) | 3883 (42.9) |  | 2398 (43.6) | 3949 (43.8) |  |
| Female | 2964 (54.8) | 5158 (57.1) |  | 3105 (56.4) | 5066 (56.2) |  |
| Body mass index, mean (SD), kg/m^2^ | 24.4 (3.6) | 24.0 (3.9) | 0.12 | 24.4 (3.6) | 24.2 (4.0) | 0.04 |
| Smoking status, No. (%) |  |  | 0.13 |  |  | 0.006 |
| Never smoker | 4862 (89.9) | 7732 (85.5) |  | 4813 (87.5) | 7867 (87.3) |  |
| Ever smoker | 547 (10.1) | 1309 (14.5) |  | 690 (12.5) | 1148 (12.7) |  |
| Comorbidities, No. (%) |  |  |  |  |  |  |
| Diabetes | 830 (15.3) | 2003 (22.2) | –0.18 | 1118 (20.3) | 1789 (19.9) | 0.01 |
| Hypertension | 1223 (22.6) | 2868 (31.7) | –0.21 | 1613 (29.3) | 2559 (28.4) | 0.02 |
| Dyslipidemia | 1726 (31.9) | 3550 (39.3) | –0.15 | 2148 (39.0) | 3334 (37.0) | 0.04 |
| Peripheral vascular disease | 373 (6.9) | 1179 (13.0) | –0.21 | 575 (10.5) | 969 (10.8) | –0.01 |
| Chronic pulmonary disease | 1025 (19.0) | 4713 (52.1) | –0.74 | 2235 (40.6) | 3595 (39.9) | 0.01 |
| Chronic liver disease | 1002 (18.5) | 2212 (24.5) | –0.15 | 1315 (23.9) | 2042 (22.7) | 0.03 |
| Chronic kidney disease | 32 (0.6) | 111 (1.2) | –0.07 | 59 (1.1) | 90 (1.0) | 0.008 |
| Malignancy | 273 (5.1) | 768 (8.5) | –0.14 | 399 (7.3) | 652 (7.2) | 0.001 |
| Charlson Comorbidity Index, mean (SD) | 1.1 (1.3) | 2.0 (1.6) | 0.52 | 1.7 (1.6) | 1.7 (1.6) | 0.06 |
| Income level, No. (%) |  |  | 0.18 |  |  | 0.03 |
| Q1 (lowest) | 1480 (27.4) | 2010 (22.2) |  | 1337 (24.3) | 2165 (24.0) |  |
| Q2 | 1302 (24.1) | 2832 (31.3) |  | 1562 (28.4) | 2558 (28.4) |  |
| Q3 | 1437 (26.6) | 2511 (27.8) |  | 1504 (27.3) | 2483 (27.5) |  |
| Q4 (highest) | 1118 (20.7) | 1617 (17.9) |  | 1054 (19.2) | 1723 (19.1) |  |
| Missing | 72 (1.3) | 71 (0.8) |  | 46 (0.8) | 85 (1.0) |  |
| Hospital size, No. (%) |  |  | 0.34 |  |  | 0.05 |
| <500 beds | 3832 (70.8) | 7025 (77.7) |  | 4150 (75.4) | 6823 (75.7) |  |
| ≥500 beds | 1536 (28.4) | 1532 (16.9) |  | 1094 (19.9) | 1862 (20.7) |  |
| Missing | 41 (0.8) | 484 (5.4) |  | 258 (4.7) | 329 (3.6) |  |
| Cardiovascular drug therapy, No. (%) |  |  |  |  |  |  |
| ACE inhibitor | 0 | 4 (0.04) | –0.03 | 0 | 3 (0.03) | –0.02 |
| ARB | 10 (0.2) | 50 (0.6) | –0.06 | 13 (0.2) | 37 (0.4) | –0.03 |
| Beta-blocker | 15 (0.3) | 66 (0.7) | –0.06 | 26 (0.5) | 54 (0.6) | –0.02 |
| Calcium-channel blocker | 19 (0.4) | 129 (1.4) | –0.11 | 54 (1.0) | 93 (1.0) | –0.005 |
| Diuretic | 19 (0.4) | 93 (1.0) | –0.08 | 35 (0.6) | 70 (0.8) | –0.02 |
| Statin | 12 (0.2) | 72 (0.8) | –0.08 | 31 (0.6) | 52 (0.6) | –0.002 |
| Insulin | 12 (0.2) | 72 (0.8) | –0.08 | 30 (0.5) | 52 (0.6) | –0.005 |
| Other hypoglycemic agents | 16 (0.3) | 82 (0.9) | –0.08 | 41 (0.8) | 64 (0.7) | 0.004 |
| Antiplatelet agents | 6 (0.1) | 57 (0.6) | –0.09 | 19 (0.3) | 39 (0.4) | –0.02 |
| Organ dysfunction, No. (%) |  |  |  |  |  |  |
| Cardiovascular | 56 (1.0) | 239 (2.6) | –0.12 | 95 (1.7) | 186 (2.1) | –0.03 |
| Respiratory | 532 (9.8) | 1566 (17.3) | –0.22 | 890 (16.2) | 1358 (15.1) | 0.03 |
| Neurologic | 13 (0.2) | 58 (0.6) | –0.06 | 23 (0.4) | 45 (0.5) | –0.01 |
| Hematologic | 13 (0.2) | 38 (0.4) | –0.03 | 18 (0.3) | 31 (0.4) | –0.003 |
| Hepatic | 1 (0.02) | 12 (0.1) | –0.04 | 1 (0.02) | 8 (0.1) | –0.03 |
| Renal | 10 (0.2) | 73 (0.8) | –0.09 | 44 (0.8) | 52 (0.6) | 0.03 |
| Metabolic | 0 | 8 (0.1) | –0.04 | 0 | 5 (0.06) | –0.03 |
| Vasopressor use, No. (%) | 52 (1.0) | 224 (2.5) | –0.12 | 77 (1.4) | 174 (1.9) | –0.04 |
| Oxygen therapy, No. (%) |  |  |  |  |  |  |
| No oxygen | 4878 (90.2) | 7499 (82.9) | 0.21 | 4617 (83.9) | 7674 (85.1) | –0.03 |
| Supplemental oxygen | 527 (9.7) | 1541 (17.0) | –0.22 | 882 (16.0) | 1340 (14.9) | 0.03 |
| High-flow nasal cannula | 114 (2.1) | 102 (1.1) | 0.08 | 94 (1.7) | 157 (1.7) | –0.002 |
| Mechanical ventilation | 46 (0.9) | 85 (0.9) | –0.01 | 59 (1.1) | 99 (1.1) | –0.003 |
| Renal replacement therapy, No. (%) | 7 (0.1) | 33 (0.4) | –0.05 | 16 (0.3) | 25 (0.3) | 0.003 |
| ECMO, No. (%) | 5 (0.1) | 2 (0.02) | 0.03 | 2 (0.04) | 2 (0.02) | 0.008 |

*ACE* angiotensin-converting enzyme, *ARB* angiotensin II receptor blocker, *COVID-19* coronavirus disease 2019, *ECMO* extracorporeal membrane oxygenation, *SMD* standardized mean difference

**Table S13** Subgroup analyses of the risk of cardiovascular outcomes in participants hospitalized for COVID-19 or non-COVID-19 pneumonia, in cohorts that included only patients with body mass index and smoking status data

| Subgroup | Hazard ratio (95% CI) - COVID-19 vs Non-COVID-19 pneumonia | | | | | | | | |
| --- | --- | --- | --- | --- | --- | --- | --- | --- | --- |
|  | MACE^a^ | All-cause mortality | Cerebrovascular disease | Dysrhythmia | Inflammatory heart disease | Myocardial infarction | Congestive heart failure | Cardiac arrest | Thrombotic disease |
| **Body mass index, kg/m^2b^** | | | | | | | | | |
| <25.0 | 0.47  (0.22–1.02) | 0.63  (0.44–0.90) | 0.61  (0.21–1.76) | 1.17  (0.66–2.06) | 1.35  (0.15–12.41) | 0.39  (0.14–1.03) | 0.23  (0.15–0.35) | 0.66  (0.18–2.38) | 0.86  (0.39–1.92) |
| ≥25.0 | 0.65  (0.31–1.33) | 1.13  (0.52–2.45) | 1.57  (0.47–5.27) | 5.34  (2.26–12.61) | N/A | 0.49  (0.21–1.17) | 0.42  (0.27–0.64) | N/A | 1.46  (0.74–2.89) |
| **Smoking status** | | | | | | | | | |
| Never smoker | 0.63  (0.36–1.11) | 0.78  (0.56–1.09) | 1.27  (0.53–3.06) | 2.08  (1.28–3.36) | 0.79  (0.09–7.34) | 0.45  (0.22–0.91) | 0.33  (0.24–0.44) | 0.80  (0.20–3.17) | 1.13  (0.68–1.88) |
| Ever smoker | 0.27  (0.06–1.22) | 0.05  (0.00–0.69) | 0.27  (0.04–1.93) | 14.73  (1.37–158.59) | 4.81  (0.23–102.70) | 0.40  (0.06–2.77) | 0.15  (0.05–0.43) | N/A | 2.37  (0.14–41.78) |

The hazard ratios and 95% CIs have been estimated in the inverse probability of treatment-weighted COVID-19 (n = 5503) and non-COVID-19 pneumonia (n = 9015) groups

*CI* confidence interval, *COVID-19* coronavirus disease 2019, *MACE* major adverse cardiovascular event, *N/A* not applicable

^a^ A composite of myocardial infarction and stroke

^b^ The cutoff value for the obese category was based on the World Health Organization guidelines for the Asia-Pacific region

**Table S14** Comparison of the risk of cerebrovascular outcomes, identified using only the International Classification of Diseases, 10th Revision codes, in participants hospitalized for COVID-19 or non-COVID-19 pneumonia

| Outcomes | No. of events | | Incidence per 1000000 person-days | | Hazard ratio  (95% CI) | *P* value |
| --- | --- | --- | --- | --- | --- | --- |
|  | COVID-19  (n = 125810) | Non-COVID-19 pneumonia  (n = 28492) | COVID-19 | Non-COVID-19 pneumonia |  |  |
| Stroke | 687 (0.6) | 383 (1.3) | 26.0 | 50.0 | 0.63 (0.55–0.72) | <0.001 |
| Ischemic | 506 (0.4) | 302 (1.1) | 19.0 | 40.0 | 0.57 (0.49–0.67) | <0.001 |
| Hemorrhagic | 127 (0.1) | 63 (0.2) | 4.9 | 8.4 | 0.65 (0.47–0.89) | 0.008 |
| TIA | 222 (0.2) | 87 (0.3) | 8.5 | 11.0 | 1.18 (0.90–1.54) | 0.24 |

The numbers of the cumulative incidence and the outcome-specific incidence rates are shown. The hazard ratios and 95% CIs have been estimated in the inverse probability of treatment-weighted COVID-19 and non-COVID-19 pneumonia groups

*CI* confidence interval, *COVID-19* coronavirus disease 2019, *TIA* transient ischemic attack
