# Supplementary material for: Cardiovascular outcomes between COVID-19 and non-COVID-19 pneumonia: a nationwide cohort study
Source: BMC Med. 2023 Oct 20;21:394. doi: 10.1186/s12916-023-03106-z (PMC10588072; doi:10.1186/s12916-023-03106-z)
Supplement: Supplementary file 3 — Additional file 3: Fig. S1. Risk of cardiovascular outcomes in participants hospitalized for COVID-19 or non-COVID-19 pneumonia, in cohorts that included patients with preexisting cardiovascular disease. Fig. S2. Subgroup analyses comparing the risk of cardiovascular outcomes in participants hospitalized for COVID-19 or non-COVID-19 pneumonia, in cohorts that included patients with preexisting cardiovascular disease. Fig. S3. Subgroup analyses comparing the risk of cardiovascular outcomes in participants hospitalized for COVID-19 or non-COVID-19 pneumonia, in cohorts that included only patients with body mass index and smoking status data. Fig. S4. Cumulative incidence of (A) MACE, (B) myocardial infarction, (C) stroke, and (D) all-cause mortality in the COVID-19 group stratified according to vaccination status. [file 12916_2023_3106_MOESM3_ESM.docx]

**Fig. S1** Risk of cardiovascular outcomes in participants hospitalized for COVID-19 or non-COVID-19 pneumonia, in cohorts that included patients with preexisting cardiovascular disease


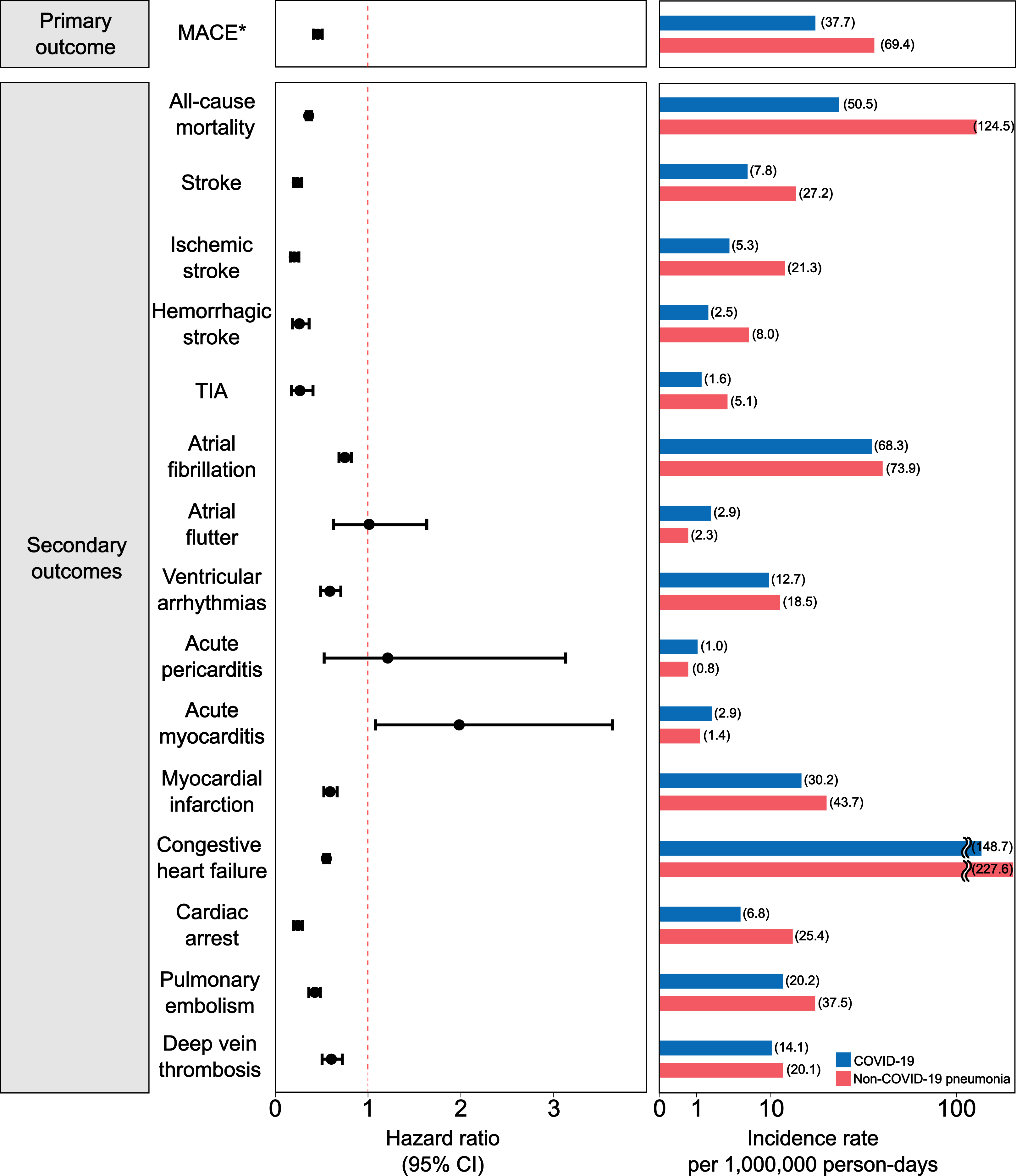


The hazard ratios and 95% CIs have been estimated in the inverse probability of treatment-weighted COVID-19 (n = 133376) and non-COVID-19 pneumonia (n = 37629) groups. The length of the bar represents the incidence rates per 1000000 person-days

*CI* confidence interval, *COVID-19* coronavirus disease 2019, *MACE* major adverse cardiovascular event, *TIA* transient ischemic attack

^*^ A composite of myocardial infarction and stroke

**Fig. S2** Subgroup analyses comparing the risk of cardiovascular outcomes in participants hospitalized for COVID-19 or non-COVID-19 pneumonia, in cohorts that included patients with preexisting cardiovascular disease.


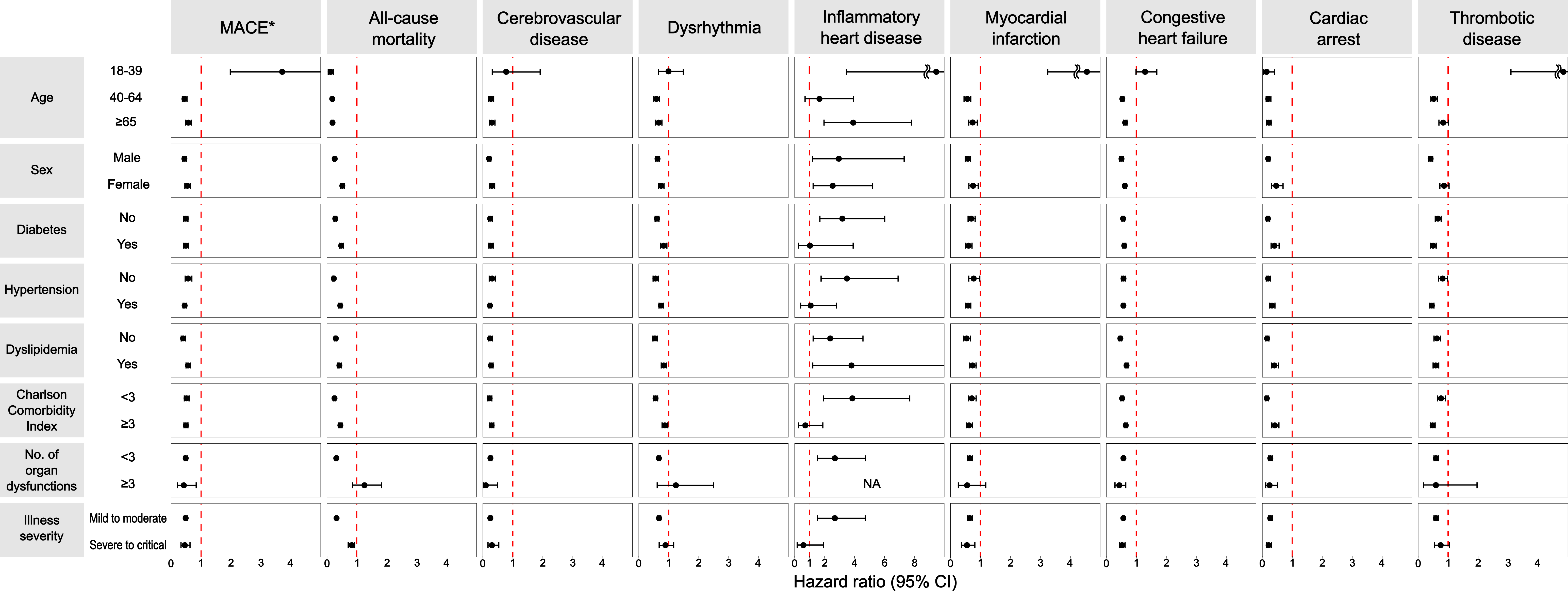


The hazard ratios and 95% CIs have been estimated in the inverse probability of treatment-weighted COVID-19 (n = 133376) and non-COVID-19 pneumonia (n = 37629) groups

*CI* confidence interval, *COVID-19* coronavirus disease 2019, *MACE* major adverse cardiovascular event

^*^ A composite of myocardial infarction and stroke

**Fig. S3** Subgroup analyses comparing the risk of cardiovascular outcomes in participants hospitalized for COVID-19 or non-COVID-19 pneumonia, in cohorts that included only patients with body mass index and smoking status data


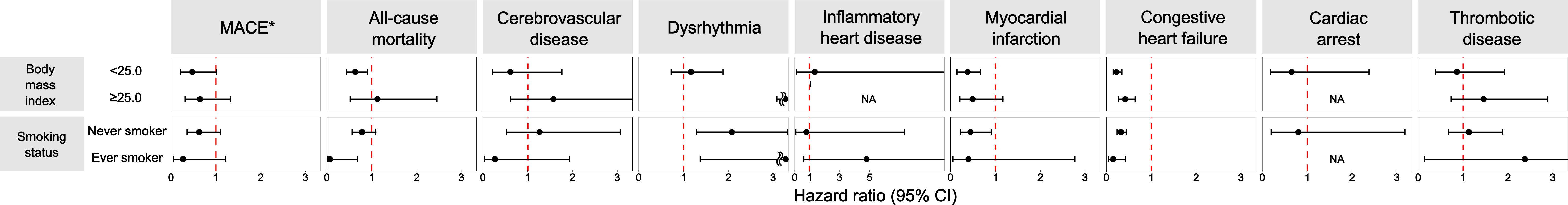


The hazard ratios and 95% CIs have been estimated in the inverse probability of treatment-weighted COVID-19 (n = 5503) and non-COVID-19 pneumonia (n = 9015) groups

*CI* confidence interval, *COVID-19* coronavirus disease 2019, *MACE* major adverse cardiovascular event

^*^ A composite of myocardial infarction and stroke

**Fig. S4** Cumulative incidence of (A) MACE, (B) myocardial infarction, (C) stroke, and (D) all-cause mortality in the COVID-19 group stratified according to vaccination status


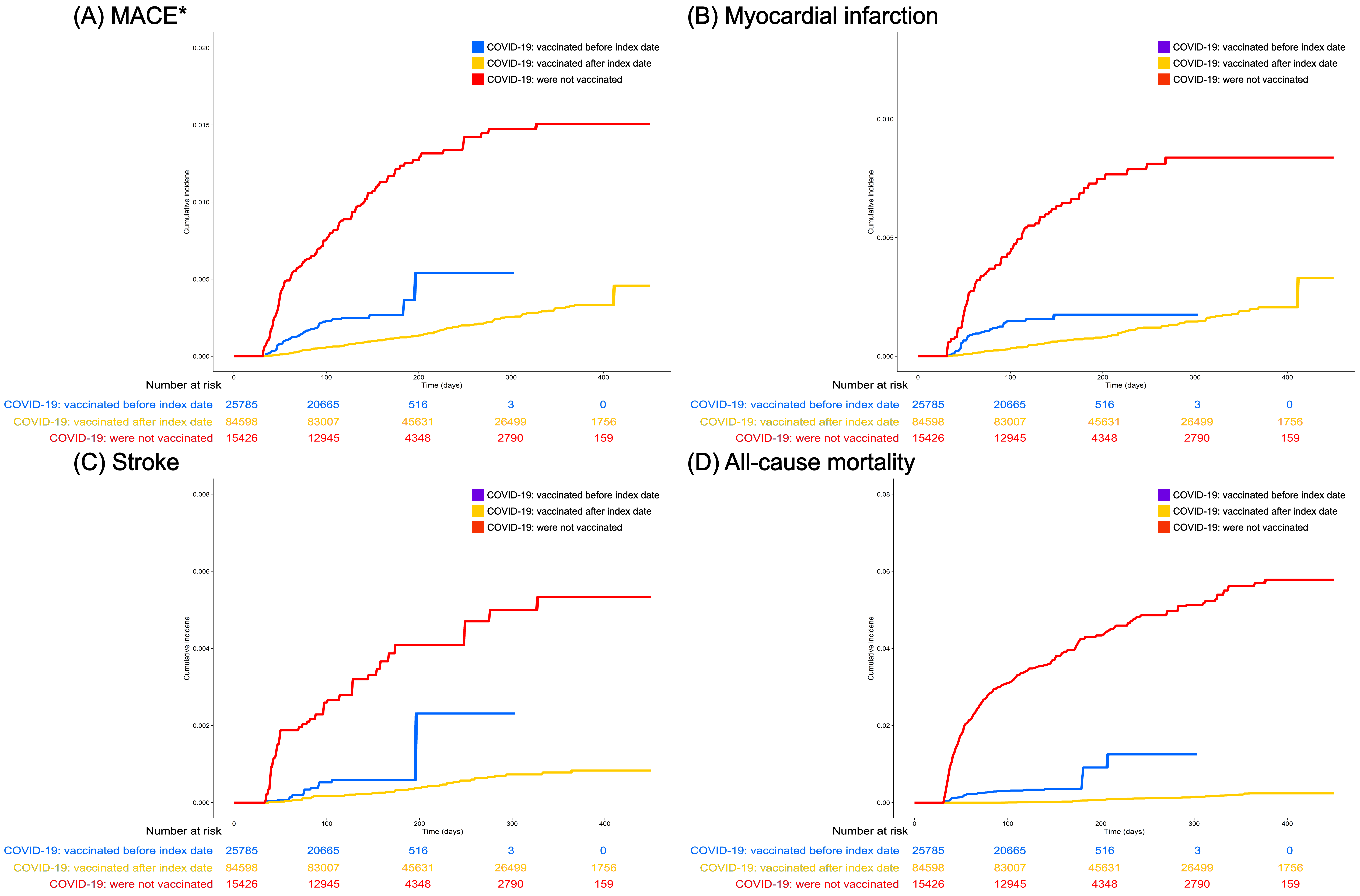


*COVID-19* coronavirus disease 2019, *MACE* major adverse cardiovascular event

^*^ A composite of myocardial infarction and stroke
